# Clinical development of CAR T cells—challenges and opportunities in translating innovative treatment concepts

**DOI:** 10.15252/emmm.201607485

**Published:** 2017-08-01

**Authors:** Jessica Hartmann, Martina Schüßler‐Lenz, Attilio Bondanza, Christian J Buchholz

**Affiliations:** ^1^ Molecular Biotechnology and Gene Therapy Paul‐Ehrlich‐Institut Langen Germany; ^2^ Division of Medical Biotechnology Paul‐Ehrlich‐Institut Langen Germany; ^3^ German Cancer Consortium (DKTK) Heidelberg Germany; ^4^ Innovative immunotherapies Ospedale San Raffaele Milano Italy

**Keywords:** ATMPs, cancer, immunotherapy, regulatory issues, toxicities, Cancer, Genetics, Gene Therapy & Genetic Disease, Immunology

## Abstract

Chimeric antigen receptor (CAR) T cell therapy, together with checkpoint inhibition, has been celebrated as a breakthrough technology due to the substantial benefit observed in clinical trials with patients suffering from relapsed or refractory B‐cell malignancies. In this review, we provide a comprehensive overview of the clinical trials performed so far worldwide and analyze parameters such as targeted antigen and indication, CAR molecular design, CAR T cell manufacturing, anti‐tumor activities, and related toxicities. More than 200 CAR T cell clinical trials have been initiated so far, most of which aim to treat lymphoma or leukemia patients using CD19‐specific CARs. An increasing number of studies address solid tumors as well. Notably, not all clinical trials conducted so far have shown promising results. Indeed, in a few patients CAR T cell therapy resulted in severe adverse events with fatal outcome. Of note, less than 10% of the ongoing CAR T cell clinical trials are performed in Europe. Taking lead from our analysis, we discuss the problems and general hurdles preventing efficient clinical development of CAR T cells as well as opportunities, with a special focus on the European stage.

**Glossary**
Advanced therapy medicinal product (ATMP): A subclass of medicinal products encompassing cell therapy, gene therapy, and tissue engineering. CAR T cells belong to this group as well. Specific legislation for ATMPs is valid in the EU.
Chimeric antigen receptor (CAR) T cells: T cells derived from the patient's own blood (autologous) or derived from a healthy person (allogenic) genetically engineered to express an artificial T cell receptor, through which they are targeted to disease‐related cells independently of MHC engagement.
Clinical end points and surrogate end points: There are multiple ways to approach clinical or surrogate end points, and individual trials may use different definitions. According to guidelines of the International Council for Harmonisation of Technical Requirements for Pharmaceuticals for Human Use (ICH), a clinical end point is a study variable to assess the clinically relevant effect of the investigational medicinal product (IMP) in a particular disease, whereas a surrogate end point relates to a clinically important outcome but does not itself measure a clinical benefit (ICH E8—general considerations for clinical trials).
*Clinical response* (*as defined by the Response Evaluation Criteria In Solid Tumors* (*RECIST*) *guideline*) The disappearance of all clinical evidence of a disease is called complete response (CR), whereas at least 30% tumor reduction is defined as partial response (PR). Less than 25% increase in tumor size is called stable disease (SD), and patients with more than 25% increasing tumor mass have progressive disease (PD).
*Overall survival* (*OS*) Time from study enrollment or randomization until death.
*Progression‐free survival* (*PFS*) Time from enrollment or randomization until disease progression or death.
*Event‐free survival* (*EFS*) Time from enrollment or randomization until disease progression, death, or discontinuation of treatment.
*Duration of response* (*DoR*) Time from confirmation of a response (CR, PR, or SD) until disease progression. Notably, clinical response is often used as surrogate end point in oncology trials, whereas improvement in survival is considered a direct measure of clinical benefit.
European Medicines Agency (EMA): European authority responsible in the European Union for evaluating marketing authorisations of medicinal products including CAR T cells submitted through the centralized procedure.
Good manufacturing practice (GMP): Production of medicinal products under defined high‐quality standards.
Toxicity associated with CAR T cell therapy: *On‐target/off‐tumor toxicity* Side effects caused by killing of healthy tissue by CAR T cells due to target antigen expression outside tumor tissue.
*Off‐target toxicity* Side effects in CAR T cell‐treated patients due to cross‐reactivity of the engineered antigen binding domain with a non‐related surface protein.
*Cytokine‐release syndrome* (*CRS*) Systemic inflammatory response resulting in non‐infective fever with elevated levels of inflammatory cytokines such as interleukin‐6 and interferon‐γ.
*Neurotoxicity* Presence of neurocognitive deficits.

## Introduction

**Figure 1:**
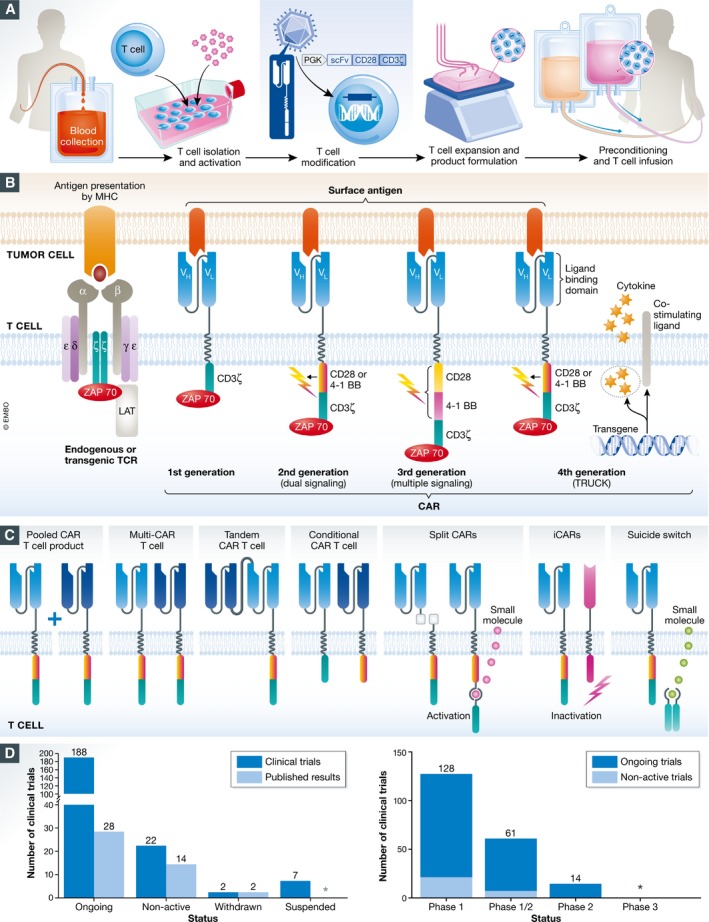
CAR T cell therapy—principle and clinical trial overview (A) The CAR T cell therapy process. T cells are isolated from blood of the patient or a donor, activated, and then genetically engineered to express the CAR construct (an example shown in gray above the vector particle in violet). After *ex vivo* expansion of the CAR T cells, they are formulated into the final product. The patient undergoes either a conditional chemotherapy or the CAR T cell product is directly infused. (B) Schematic representation of a T cell receptor (TCR) and four types of chimeric antigen receptors (CARs) being displayed on the surface of a T cell while contacting their antigen (red) on the tumor cell. The single‐chain variable fragment (scFv) as ligand‐binding domain mediating tumor cell recognition in CARs is shown in light blue with the VH and VL domains being connected via a long flexible linker and transmembrane domain to intracellular signaling domains. Pro‐inflammatory cytokines or co‐stimulatory ligands expressed by the CAR T cells are depicted for the 4^th^ generation. (C) Overview of so‐called smart CAR T cells products. Pooled CAR T cell products consist of two or more single‐targeting CAR T cell types with distinct antigen specificities. Multi‐CAR T cells harbor several CAR molecules with different antigen specificities. A tandem CAR T cell expresses a CAR construct harboring two ligand‐binding domains with different antigen specificities. In a conditional CAR T cell activation and co‐stimulation are separated on two CAR constructs recognizing different target antigens. In the split CAR construct the ligand‐binding or signaling domain is physically separated allowing controlled CAR T cell activation. iCAR T cells additionally express a receptor engineered to recognize an antigen expressed on normal tissue to provide an inhibitory signal in turn. In addition CAR T cells can be equipped with suicide genes or switches (e.g., iCasp9) allowing ablation of CAR T cells. (D) Left, status of published CAR T cell gene therapy trials or trials registered at ClinicalTrials.gov including long‐term follow‐up studies. The status of one trial is unknown and not listed. The total number of clinical trials (dark blue bars) is compared to published clinical trials (light blue bars). The asterisk indicates zero trials. Right, phases of CAR T cell gene therapy trials. Long‐term follow‐up studies are not included. For nine trials, the phase classification is unknown. The asterisk indicates zero trials.

For many decades, cancer therapy mainly relied on surgery, chemotherapy, and radiotherapy. In recent years, the concept of stimulating the patient's immune response and the observed durability of responses has established cancer immunotherapies as a novel treatment option for a series of cancer types. One promising approach is the adoptive transfer of T cells genetically engineered to express a chimeric antigen receptor (CAR) (Fig 1A). Such CAR T cells recognize surface antigens independently from MHC restriction. When targeted to tumor surface antigens, CAR T cells proliferate and kill tumor cells upon antigen contact (Fesnak *et al*, 2016).

CARs are composed of an extracellular binding domain, a hinge region, a transmembrane domain, and one or more intracellular signaling domains (Fig 1B). Single‐chain variable fragments (scFvs) derived from tumor antigen‐reactive antibodies are commonly used as extracellular binding domains. All CARs harbor the CD3ζ chain domain as the intracellular signaling domain. Second‐ or third‐generation CARs also contain co‐stimulatory domains, like CD28 and/or 4‐1BB, improving proliferation, cytokine secretion, resistance to apoptosis, and *in vivo* persistence. Third‐generation CARs exhibit improved effector functions and *in vivo* persistence as compared to second‐generation CARs, whereas fourth‐generation CARs, so‐called TRUCKs or armored CARs, combine the expression of a second‐generation CAR with factors that enhance anti‐tumoral activity, such as cytokines, co‐stimulatory ligands, or enzymes that degrade the extracellular matrix of solid tumors (Fig 1B; Chmielewski & Abken, 2015). To enhance the safety of CAR T cell therapy, so‐called smart T cells which are either equipped with a suicide gene or include synthetic control devices are under non‐clinical and clinical investigation (Fig 1C; Zhang & Xu, 2017).

Thus, CAR T cells are complex medicinal products with the unique feature of being able to self‐amplify and persist in treated patients. Their translation from basic and pre‐clinical research to clinical trials therefore poses many challenges that slow down clinical development, while many cancer patients desperately await novel treatment options. With the aim of identifying the hurdles in clinical translation of this therapeutic concept, we have analyzed all available data from ongoing and completed clinical trials. Based on our analysis, we offer suggestions to facilitate translation of CAR T cell products especially in Europe.

## Completed and ongoing CAR T cell clinical trials

As of the end of 2016, 220 CAR T cell trials are documented of which 188 are ongoing including nine long‐term follow‐up studies (Fig 1D, Datasets EV1 and EV2, Appendix Table S1). Most of the clinical trials conducted are phase 1 (128) primarily evaluating safety and dose finding, but phase 1/2 and phase 2 trials assessing efficacy are catching up especially with CD19 as the CAR antigen (39 of 75 phase 1/2 or phase 2 trials; Fig 1D, Datasets EV1 and EV2).

**Figure 2:**
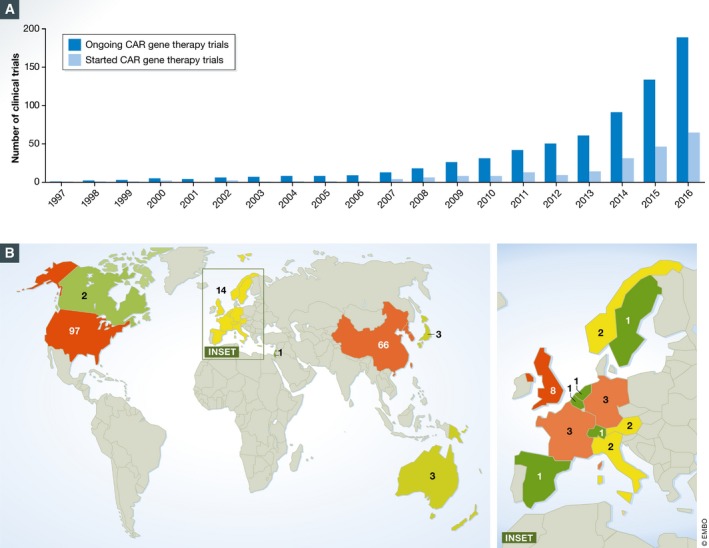
CAR T cell trials over time and geographical distribution (A) Timeline of cancer CAR T cell trials as listed in Datasets EV1 and EV2 distinguishing between ongoing number (dark blue bars) and newly initiated trials in the indicated year (light blue bars). (B) Geographical distribution of worldwide ongoing CAR T cells clinical trials (left) and distribution of trial sites of the ongoing European studies (right). Five studies are multi‐centric, of which four are multi‐country trials in Europe (Dataset EV5). Long‐term follow‐up studies are not included. Color code indicates the prevalence of trials from low (green) to high (red).

The first CAR T cell trials initiated about 20 years ago included patients with advanced epithelial ovarian cancer or metastatic renal cell carcinoma and targeted the folate receptor or carbonic anhydrase IX (CAIX), respectively (Kershaw *et al*, 2006; Lamers *et al*, 2006). The next two registered clinical trials with published results reported on single patients suffering from neuroblastoma (Dataset EV3) or follicular lymphoma (Dataset EV4) reaching complete response (Park *et al*, 2007; Till *et al*, 2008). However, the breakthrough was achieved over the following years with CD19‐specific CAR T cells targeting B‐cell malignancies. Complete or partial response was reported not only for single individuals but also for the majority of patients in some trials (Dataset EV4). From then on, the number of CAR T cell trials substantially increased and now grows exponentially (Fig 2A). In 2016 alone, 62 new CAR T cell clinical trials have been entered into ClinicalTrials.gov.

CAR T cell therapy was initially introduced in the USA, then spreading to the rest of the world (Fig 2B). Currently, 89 CAR T cell clinical trials are in progress outside the USA, with highest numbers in China (66 trials) and Europe (14 trials; Dataset EV5). Compared to the USA and China however, Europe is clearly lagging behind. The majority of trials in Europe are performed in UK (8), followed by Germany (3) and France (3) (Fig 2B).

**Figure 3:**
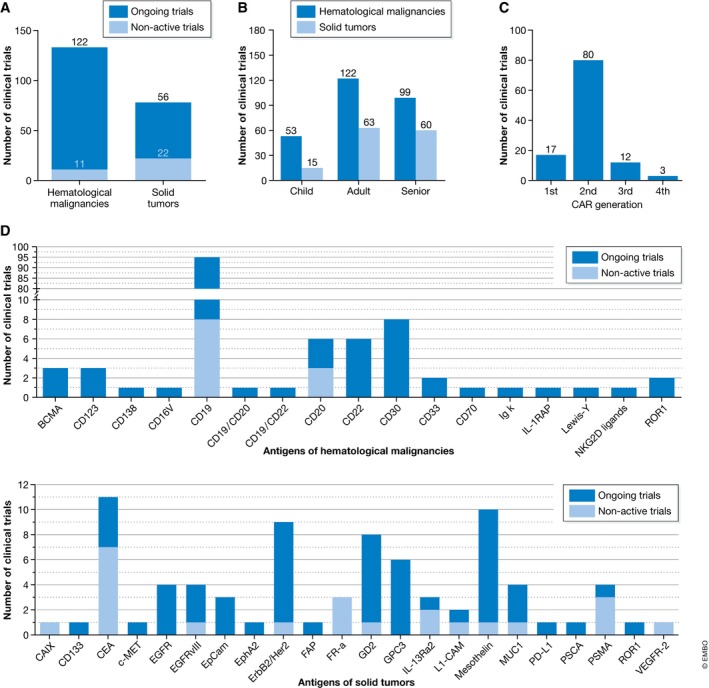
Indication, age, CAR generation, and targeted antigen in clinical trials (A) Solid tumors versus tumors of the hematopoietic and lymphoid system. The number of ongoing trials (dark blue bar) is compared to the number of non‐active trials (light blue bar). (B) Patient age distribution for solid tumors (light blue bars) and hematological malignancies (dark blue bars). (C) Generation of the CAR constructs applied. (D) Targeted antigens separated for tumors of hematopoietic or lymphoid origin (upper panel) and for solid tumors (lower panel).

Of the current trials, 133 target hematological malignancies and 78 solid tumors (Fig 3A and B; Datasets EV1 and EV2). For tumors of the hematopoietic and lymphoid system, 17 different CAR antigens are under investigation (Fig 3D). The most frequently targeted antigen is CD19 with 56 ongoing and eight non‐active trials. Even more antigens (22) are investigated for the treatment of solid tumors (Fig 3D). Previous trials focused on CEA as antigen‐targeting colorectal cancer, breast cancer, gastric cancer, adenocarcinoma as well as liver metastases. Ongoing trials target mesothelin, ErbB2/Her2, GD2 (neuroblastoma or sarcoma), or GPC3 (hepatocellular carcinoma).

Most clinical trials have used autologous, unselected peripheral blood mononuclear cells (PBMC) as the starting material and IL‐2 for stimulation resulting in a CAR T cell product consisting of CD4 and CD8 T cells with an activated effector T‐cell phenotype. More recently, methods to isolate defined T cell subsets or to drive T cells into a certain phenotype have been developed (Xu *et al*, 2014; Ramos *et al*, 2016; Turtle *et al*, 2016). In addition, automated manufacturing might be an option to simplify the process and enhance the robustness of CAR T cell production. CD19‐CAR T cells generated using a closed automated GMP cell processing system have been shown to be comparable to CD19‐CAR T cells produced by the conventional processes in terms of transduction efficiency, phenotype, function, and overall yield (Mock *et al*, 2016; Priesner *et al*, 2016).

**Figure 4:**
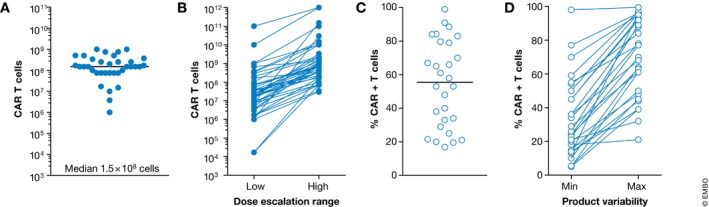
CAR T cell dose and percentages of CAR‐positive cells within CAR T cell products (A, B) CAR T cell dose indicated in the study description of published CAR T cell gene therapy trials or trials registered at ClinicalTrials.gov. The CAR T cell dose is normalized to 75 kg or 1.72 m^2^ per dose. CAR T cells are either administered as a fixed dose (A) or in a dose escalation regimen (B). Each dot represents a single trial. (C, D) The reported number of CAR‐positive cells as given in Datasets EV3 and EV4 in column “%CAR^+^ cells (median; range)” was used to identify the median amount of CAR‐positive T cells in the various cell products within one clinical trial (C) and the range of variability between cell products within one clinical trial (lowest (min) and highest (max) percentage CAR‐positive T cells per trial) (D).

Typically, CAR T cells are infused intravenously. However, intra‐tumoral (You *et al*, 2016), intracranial (Brown *et al*, 2015) or intra‐peritoneal injection (Koneru *et al*, 2015), hepatic artery (Katz *et al*, 2015), pleural (Petrausch *et al*, 2012), or transcatheter arterial infusion are being investigated as well (Datasets EV1 and EV2). To increase the tolerability of the treatment and to lower the risk of side effects, the given CAR T cell dose is often split over multiple injections (e.g., three injections each 1 day apart; Datasets EV3 and EV4). The total treatment dose is in the range of 7.5 × 10^7^–3.4 × 10^8^ CAR T cells if a fixed dose is applied (Fig 4A). However, the majority of trials use an inter‐ or intra‐patient dose escalation regime (Datasets EV1 and EV2). Dose escalation usually covers 2‐log steps starting somewhere between 1 × 10^6^ and 1 × 10^9^ CAR T cells (Fig 4B). Notably, the total number of infused cells depends on the percentage of CAR‐positive T cells within the product, which is highly variable, not only between different studies (Fig 4C) but also within single trials (Fig 4D and Datasets EV3 and EV4).

For the generation of CAR T cells, a slight preference for the use of gamma‐retroviral vectors (RVs), directly followed by lentiviral vectors (LVs), can be observed. Only very few clinical studies used electroporation for the transfer of the CAR construct (Datasets EV3 and EV4). In the majority of all trials, second‐generation CARs were transferred (Fig 3C). Third‐ or fourth‐generation CARs are being tested especially when targeted to CD19 (Dataset EV1).

## Clinical benefit for many cancer patients

The most famous case of a patient who benefitted from CAR T cell therapy is probably that of Emily Whitehead, a child suffering from recurrent acute lymphoblastic leukemia (ALL), who has now reached 5 years of cancer‐free survival (http://emilywhitehead.com). Emily was part of the NCT01626495 trial, and thus, one of the more than 300 patients with hematological malignancies in addition to the about 150 patients with solid tumors for which published data are available (Datasets EV3 and EV4).

**Figure 5:**
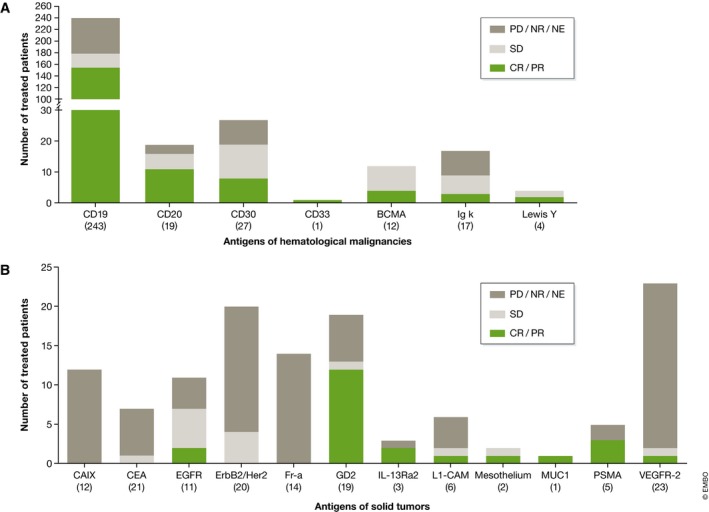
Clinical outcome (A, B) Best clinical outcome for hematological malignancies (A) and solid tumors (B) dependent on the targeted antigen. The number of treated patients is provided in brackets below the targeted antigen. CR, complete response; PR, partial response; SD, stable disease; PD, progressive disease; NR, no response; NE, not evaluable.

CAR T cell therapy appears to be especially active against B‐cell malignancies. This is due to the tumor cell selective and homogenous expression of CD19 or CD20 as well as the easier access for CAR T cells. Taking into account the dismal prognosis for late‐stage patients, even when balanced against the observed toxicities (see below), the clinical response observed in CAR T cell trials treating CD19‐positive malignancies is substantial. From the 243 patients (199 adult, 44 pediatric) treated with CD19‐CAR T cells, objective response has been observed for more than 60% while only 20% did not respond (Fig 5A, Dataset EV4). Of note, in trials including pediatric and adult patients, the clinical outcome appeared to be independent from age (Cruz *et al*, 2013; Maude *et al*, 2014; Lee *et al*, 2015; Zhang *et al*, 2016). Whether this holds true for overall survival will be established once long‐term monitoring data become available.

In five trials, more than 85% of treated patients reached complete response (CR) as best clinical outcome (NCT00968760, NCT01865617, NCT01815749, NCT01626495, NCT01044069) (for details, see Dataset EV4). In these trials, the time points for evaluation range from 4 weeks (Turtle *et al*, 2016) to 30 months (Kebriaei *et al*, 2016). They included patients suffering from ALL or non‐Hodgkin lymphoma (NHL) with different grades of detectable disease in bone marrow (BM), extramedullary sites, or cerebrospinal fluid. Patients with minimal residual disease (MRD) or morphologic disease, defined by the percentage of blasts in BM, were also included (Dataset EV4). Interestingly, there appears to be no major difference in remission rates between patients with morphologic disease and patients with MRD (Davila *et al*, 2014; Maude *et al*, 2014; Turtle *et al*, 2016; Park *et al*, 2017). However, patients with MRD lived significantly longer (18‐month follow‐up) (Park *et al*, 2017). Thus, low disease burden seems to improve the durability of CAR T cell therapy, at least in ALL.

Chemotherapy can be applied to reduce tumor burden before CAR T cell therapy. After CAR T cell therapy, patients who achieved a complete response can be offered allogeneic hematopoietic stem cell transplantation (HSCT) to provide additional curative potential. In the ALL studies, the remissions induced by CAR T cells have been variously consolidated with transplantation [three out of 30, 11%; (Maude *et al*, 2014); seven out of 14, 50% (Davila *et al*, 2014); 10 out of 14, 71% (Lee *et al*, 2015); 13 out of 27, 48% (Turtle *et al*, 2016)]. Despite these differences and with all the limitations of short‐term follow‐up, the durability of responses between the studies appears to be remarkably similar. This suggests that CAR T cells might provide a substantial clinical benefit regardless of HSCT consolidation. Moreover, it is often overlooked that in the same studies, a substantial fraction of patients going into remission after CAR T cells had been previously transplanted, indicating that diseases insensitive to the graft‐versus‐leukemia effect can be instead sensitive to engineered T cells.

Overall, there is a tendency for CD19‐CAR T cells to be most effective in patients suffering from ALL, slightly less so for NHL, and the least for chronic lymphocytic leukemia (CLL), suggesting an influence of the disease type on efficacy. Although preliminary, the results of CAR T cells in NHL appear promising. A mixed patient population included nine high‐grade chemorefractory NHL cases, of which four achieved complete remission and additional two partial remission (Kochenderfer *et al*, 2015). The initial result of a subsequent trial sponsored by Kite Pharma with the same CAR T cell product confirmed these results, with four out of seven patients treated achieving complete remission, which is continuing after 12 months (Locke *et al*, 2017). Considering that the prognosis of high‐grade chemorefractory NHL is particularly dismal with a median survival of few weeks, these results prompted filing of the Kite Pharma CAR T cell product with the Food and Drug Administration (FDA). For other tumor antigens of hematological malignancies, clinical benefit is less pronounced (Fig 5A, Dataset EV4), although final results from these mostly ongoing trials are still to come.

At variance with the results obtained for hematological malignancies, no encouraging data have been published for solid tumors (12 different antigens targeted), besides anecdotal evidence for remissions in single patients (Fig 5B, Dataset EV3). An exception is the case of CAR T cells specific for GD2 which resulted in more than 50% CR in a phase I clinical trial addressing neuroblastoma patients (Louis *et al*, 2011).

The high remission rates obtained with CD19‐CAR T cells in B‐ALL compare favorably to standard chemotherapy as well as to recently approved antibody‐based therapies, such as blinatumomab, a bispecific T cell engager (BiTE) directed against CD3 and CD19. Blinatumomab induced complete remission in 42.9% of patients with Philadelphia chromosome‐negative relapsed or refractory ALL, with a median relapse‐free survival of 5.9 months according to the European public assessment report (EPAR; EMA/CHMP/469312/2015). The single‐arm phase II study was the basis for conditional marketing authorization in the EU. The favorable outcome and superior efficacy of blinatumomab (34% complete remission rate at 12 weeks, median OS 7.7 months) over standard chemotherapy (16% CR rate, 4.0‐month median OS) have been confirmed in a randomized controlled trial (Kantarjian *et al*, 2017). Direct comparison of complete remission rates between blinatumomab and CAR T cells in B‐ALL is misleading, however, due to differences in time points of outcome assessment (12 weeks in the case of blinatumomab and 28 days in the case of CAR T cells). Yet, the reported durable remissions and event‐free survival rates following CAR T cell administration are particularly promising, especially since these patient populations included cases refractory to blinatumomab (Maude *et al*, 2014). Very recently, Novartis announced that the corresponding CAR T cell product CTL019 (tisagenlecleucel) received recommendation for approval by the FDA Oncologic Drugs Advisory Committee for the treatment of relapsed or refractory pediatric and young adult patients with B‐cell ALL (www.novartis.com/news/media-releases/novartis-car-t-cell-therapy-ctl019-unanimously-10-0-recommended-approval-fda). The final decision about marketing approval will be taken by the FDA and is expected within the next months.

As can be easily verified in the examples listed above, CAR T cell trials differ in many parameters including disease entity, disease burden, CAR construct design, production and amplification of CAR T cells, patient pre‐conditioning and administered doses, to name just a few. Given this complexity, the identification of the most relevant parameters for a positive clinical outcome is the prime focus of ongoing research. In those CD19‐CAR T cell trials which resulted in substantial benefit for the patients, second‐generation CARs were usually used (Dataset EV4). However, many other CD19‐CAR T cell trials that also used second‐generation CARs showed a less promising outcome. Obviously, other still unknown parameters impair a positive outcome.

Lymphodepletion was demonstrated to be beneficial for enhanced *in vivo* CAR T cell expansion and persistence (Dai *et al*, 2015; Turtle *et al*, 2016), while IL‐2 co‐administration was not recommended (Zhang *et al*, 2015). Another important factor influencing *in vivo* expansion is the CAR T cell phenotype. Products containing higher amounts of CAR T cells with a central (CD62L^+^) and/or stem cell memory phenotype (CD45RA^+^) showed enhanced *in vivo* expansion (Xu *et al*, 2014). Notably, this phenotype can be preserved during manufacturing by using IL‐7 and IL‐15 instead of IL‐2 for cultivation (Casucci *et al*, 2013). Such CAR T cells may become less exhausted after repeated antigen‐specific stimulation (Xu *et al*, 2014). In a similar direction, administration of a defined CD4:CD8 CAR T cell composition (ratio 1:1) showed for the first time a correlation between cell dose and the time point of peak CAR T cell expansion (Turtle *et al*, 2016). Interestingly, the absolute number of CD8^+^ CAR T cells was higher than that of CD4^+^ CAR T cells at the peak of expansion, despite CD4^+^ and CD8^+^ CAR T cells having been infused in a 1:1 ratio. Also, the used co‐stimulatory domain influences CAR T cell activity. The CD28 domain confers higher anti‐tumoral activity (Zhao *et al*, 2015), whereas longer persistence of CAR T cells is observed for the 4‐1BB domain [<1 month for CD28 (Brentjens *et al*, 2013) versus up to 4 years for 4‐1BB (Porter *et al*, 2015)].

## Severe side effects and toxicities

While CAR T cell therapy has shown impressive clinical benefit, it is sometimes associated with a variety of toxicities that can be life‐threatening (see Datasets EV3 and EV4 for published adverse events). Several death cases were reported, especially in the last year. These were due to neurotoxicity caused by cerebral edemas in the CD19‐CAR trials sponsored by Juno Therapeutics. After the first reported deaths, the trial was interrupted and the conditioning regimen was changed from cyclophosphamide and fludarabine to cyclophosphamide alone. However, soon after reinitiation, two more fatal cases excluded the conditioning regimen as causative. These fatal outcomes are on the one hand surprising when considering that other ongoing CD19‐CAR T cell trials did so far not report cumulating fatal cases of cerebral edemas (Table 1) (DeFrancesco, 2017). On the other hand, there had been at least one fatal outcome of neurotoxicity in another CD19‐CAR T trial (NCT01865617) 122 days after CAR T cell infusion (Turtle *et al*, 2016). Furthermore, reversible symptoms of neurotoxicity including confusion, delirium, expressive aphasia, encephalopathy, and seizures were reported in several other studies (Brentjens *et al*, 2011; Maude *et al*, 2014; Kochenderfer *et al*, 2015; Lee *et al*, 2015; Turtle *et al*, 2016). In some patients, CD19‐CAR T cells have been found in cerebrospinal fluid (CSF; Brentjens *et al*, 2011; Lee *et al*, 2015). Whether neurological toxicities are solely restricted to CD19‐specific CAR T cells or generally associated with CAR T cell therapy remains to be elucidated. Indeed, the potential causes for the occurrence of neurotoxicity are under debate. The postulated pathophysiological mechanisms include cytokine diffusion and/or translocation of activated CAR T cell across the blood–brain barrier.

**Table 1:** Severe side effects in selected CD19‐CAR T cell trials

Identifier (Synonym)	NCT02535364 (ROCKET)	NCT02348216 (ZUMA‐1)	NCT02435849 (ELIANA)	NCT01865617	NCT01864889
Sponsor	Juno Therapeutics^a^	Kite Pharma	Novartis	FHCRC	CPLA
IMP	JCAR015	KTE‐C19	CTL019	N.A.	N.A.
CAR type	CD19/CD28/CD3z	CD19/CD28/CD3z	CD19/4‐1BB/CD3z	CD19/4‐1BB/CD3z	CD19/4‐1BB/CD3z
Indication	ALL	NHL	ALL	ALL	ALL, NHL
Included patients	N.A.	51	50	29	9
Clinical outcome	N.A.	47% CR	82% CR	90% CR	55% CR
Dose (%CAR^+^ cells)	N.A.	2 × 10^6^/kg (N.A.)	2.9 × 10^6^/kg (N.A.)	2 × 10^6^/kg (82%)	≥ 3.0 × 10^6^/kg (N.A.)
Persistence	N.A.	Up to 12 months	≥ 6 months	> 8 months	Up to 3 months
Conditioning	cy + flu or cy alone^b^	low‐dose cy + flu	cy + flu	cy + flu	Optional
Reported death cases	Three fatal cases of cerebral edema (cy + flu), two fatal cases of cerebral edema (cy alone)	Two fatal cases due to CRS	One fatal case of intracranial hemorrhage prior to disease assessment	One fatal case of irreversible neurologic toxicity 122 days after CAR T cell infusion	One fatal case of tumor lysis syndrome and one fatal case of GVHD
Neurological toxicities (grade ≥ 3)	N.A.	29% of treated patients	15% of treated patients	34% of treated patients	Not observed
References	DeFrancesco (2017), press release	Neelapu *et al* (2016), Locke *et al* (2017)	Grupp *et al* (2016)	Turtle *et al* (2016)	Dai *et al* (2015)

IMP, investigational medicinal product; ALL, acute lymphoid leukemia; NHL, non‐Hodgkin lymphoma; N.A., not available; CRS, cytokine‐release syndrome; cy, cyclophosphamide; flu, fludarabine; GVHD, graft‐versus‐host disease; SAEs, severe adverse events; CPLA, Chinese PLA General Hospital; FHCRC, Fred Hutchinson Cancer Research Center.

a Juno is using cyclophosphamide plus fludarabine pre‐conditioning treatment in other CAR T cell trials with the same or another IMP so far without reported cases of irreversible neurologic toxicities (NCT01044069, NCT01840566, NCT02028455, NCT02631044, NCT01865617). Notably, this trial has been discontinued due to neurologic toxicities.

b The conditioning regime was changed from cyclophosphamide plus fludarabine to cyclophosphamide alone upon the first death cases (DeFrancesco, 2016).

A direct connection to another frequent side effect, the cytokine‐release syndrome (CRS), appears likely. CRS has so far been the most frequent observed adverse drug reaction. The hallmark of CRS is immune activation resulting in elevated inflammatory cytokines especially IL‐6 (Lee *et al*, 2014). Symptoms such as high fever, fatigue, nausea, tachycardia/hypotension, and cardiac dysfunction have most often been reported in trials with CD19‐CARs but also occurred when other antigens of hematological malignancies, or mesothelin for the treatment of solid tumors, were targeted (Beatty *et al*, 2014). Systemic corticosteroid administration rapidly reversed symptoms in most cases (Davila *et al*, 2014; Lee *et al*, 2015), but, can result in ablation of the infused CAR T cells, thus limiting the anti‐tumoral effect (Davila *et al*, 2014). A currently preferred alternative is treatment with tocilizumab, a therapeutic IL‐6 receptor blocking antibody, which does not affect CAR T cell persistence (Davila *et al*, 2014; Maude *et al*, 2014). However, one death case has been reported due to severe CRS with multi‐organ failure 3 days after the CAR T cell infusion, despite treatment with tocilizumab, the TNFα inhibitor etanercept, and corticosteroids (Turtle *et al*, 2016).

Besides CRS and neurotoxicity, other severe complications have been observed. In contrast to the former, however, their pathogenesis is better understood and can thus be more easily prevented. Tumor lysis syndrome (TLS) results from rapid tumor cell death leading to metabolic disturbances like hyperuricemia and hyperkalemia, among others. TLS has been reported for at least four different studies treating hematological malignancies (Kochenderfer *et al*, 2013; Maude *et al*, 2014; Dai *et al*, 2015; Guo *et al*, 2016). Notably, one ALL patient died from acute TLS 12 h after receiving a second CD19‐CAR T cell infusion (Dai *et al*, 2015). Reducing tumor size before treatment and/or controlling the extent of tumor lysis by adapting the amount of infused CAR T cells can be applied to control TLS.

Both cellular and humoral immune responses resulting in the rejection of CAR T cells have been observed (Kershaw *et al*, 2006; Jensen *et al*, 2010; Turtle *et al*, 2016). This did not only limit the anti‐tumoral activity but resulted in acute anaphylaxis for some patients after the third infusion with CAR T cells harboring mouse scFv sequences (Beatty *et al*, 2014). To circumvent this unwanted immune reaction, only humanized scFv should be used in the future. To date, already seven ongoing clinical trials are using humanized CAR constructs (Datasets EV1 and EV2).

On‐target, off‐tumor recognition has become a relevant concern, since many targeted tumor antigens are also expressed on normal tissue. Among these, B‐cell aplasia is a common adverse event in CAR T cell trials targeting B‐cell malignancies (Kochenderfer *et al*, 2013; Maude *et al*, 2014; Turtle *et al*, 2016). The CD19 as well as CD20 antigen is expressed by transformed malignant as well as healthy B cells, which are therefore killed by CD19‐specific or CD20‐specific CAR T cells. The severity of B‐cell aplasia ranges from transient (Kochenderfer *et al*, 2013) to long lasting (up to 1 year reported; Maude *et al*, 2014). Notably, B‐cell aplasia is a surrogate marker, rather than a causative factor of CAR T cell activity. Patients can have a durable response even without B‐cell aplasia (Brentjens *et al*, 2011; Till *et al*, 2012), although in most trials, B‐cell aplasia correlates with clinical benefit (Maude *et al*, 2014; Porter *et al*, 2015). In addition, patients have been described with ongoing long‐term remission with recovered B‐cell counts (Kochenderfer *et al*, 2015). B‐cell aplasia can be effectively managed by infusion with gamma globulin as replacement therapy, although this is certainly costly especially in case of long‐term treatment. In addition, CD20‐CAR T cells have been reported to damage normal tissue in sites around lesions due to a low‐level expression of CD20 on normal, non‐B‐cell tissue leading to dyspnea and respiratory distress (Wang *et al*, 2014).

The most severe case of on‐target toxicity was reported in a trial targeting ErbB2 in patients suffering from lung carcinoma. Due to the recognition of ErbB2 on normal lung cells, one patient died from rapid respiratory failure and multi‐organ dysfunction (Morgan *et al*, 2010). Lowering the T‐cell dose and using second instead of third‐generation CARs may prevent this type of toxicity (Ahmed *et al*, 2015).

To improve the safety and efficacy of CAR T cell therapy, 4^th^‐generation CARs as well as smart CARs have entered clinical trials. These may overcome the obstacles encountered in the current trials, such as loss of targeted antigen, off‐target toxicity, or low persistence. For example, NCT02465983 uses a pooled CAR T cell product consisting of mesothelin‐ and CD19‐specific CAR T cells. In the clinical trial description available at ClinicalTrials.gov, the investigators hypothesize that this combination therapy may prolong the duration of mesothelin‐specific CAR T cells in the body, due to the ablation of B cells and thus CAR‐specific antibodies by the CD19‐CAR T cell. In contrast, NCT02737085 applies sequential infusion of CD19‐ and CD20‐targeted CAR T cells to reduce the risk of B‐cell relapse through a CD19 escape mutation. In the same context, NCT02903810 uses a pooled CAR T cell product consisting of CD19‐ and CD22‐specific CAR T cells. In addition, eight trials having a suicide gene or suicide switch integrated have been initiated (Datasets EV1 and EV2).

## The regulatory landscape for CAR T cells

CAR T cells combine features of cell therapy, gene therapy, and immunotherapy. In the EU, they are classified as an advanced therapy medicinal product (ATMP) and within this category, as a gene therapy medicinal product (GTMP). Until today, eight ATMPs have been granted marketing authorization in the EU, among these three are GTMPs. Two of them contain genetically modified hematopoietic stem cells (Strimvelis) or T cells (Zalmoxis) and are most closely related to CAR T cells. Although there is only one gene therapy product authorized on the US market, the oncolytic virus Imlygic, the number of ongoing CAR T cell clinical trials within the USA (89) by far outcompetes that in Europe (14) (Fig 2B).

Potential reasons for this discrepancy have recently been discussed and commented. They include lack of critical mass, gaps in knowledge and experience of translating innovative development candidates to the clinic, funding and sponsoring for clinical trials, access to manufacturing facilities, and options for founding small enterprises are only some examples (Duda *et al*, 2014; Rietschel *et al*, 2015). While a direct comparison between the situation in the USA and the EU is difficult, it is evident that many hurdles hindering a straight‐forward translation of the CAR T cell technology exist in the EU (Table 2). One of the most critical points is probably an effective infrastructure. This may appear surprising, since more than 40,000 HSCT are performed annually in Europe and in addition, significant experience and knowhow exists in initiating gene therapy trials for primary inherited immunodeficiencies (PIDs; Kohn, 2010; Passweg *et al*, 2016). However, gene therapy trials for PIDs are different in that they involve very few patients (e.g., 1 patient per 200,000 inhabitants for ADA‐SCID) which are monitored over long periods at single specialized hospitals (Cicalese & Aiuti, 2015; Whitmore & Gaspar, 2016). Accordingly, the marketing approval for Strimvelis was based on the results of a pivotal trial including a limited number of 18 children (Schimmer & Breazzano, 2016). In cancer, even when rare indications such as ALL are investigated, more than 2,500 patients are available (Sant *et al*, 2010). While the substantial amount of HSCT per year shows that large patient numbers can in general be treated, the challenge in CAR T cell therapy goes beyond that. CAR T cell therapy requires multi‐center efforts combined with high capacities in generating vector stocks and CAR T cells. The bottleneck here is a compatible infrastructure associated with hospitals providing access to large GMP facilities able to manufacture CAR T cells of high quality and consistency. This is available in only very few places in Europe.

**Table 2:** Hurdles and possible solutions for the clinical translation of CAR T cells

Hurdles	Possible solutions
Infrastructure for efficient translation missing	Support for establishing clinical centers that combine basic research, GMP production, and clinical research
CAR T cells are genetically modified organisms (GMOs) in certain EU member states and therefore require a release certificate prior to clinical evaluation	Facilitate process by putting together a universal documentation on the GMO characteristic of CAR T cells, which will then be applicable to any CAR T cell product
Different requirements among EU member states	Harmonize requirements between member states. To improve the current situation, the Voluntary Harmonization Procedure (VHP) was established (regulation 536/2014 EC)
Lack of disseminated knowledge/specific guidance	Set up databases for ATMP clinical trials and products as well as technology transfer networks
	Preparation of CAR T cell‐specific guidelines
	Early contact with national competent authorities or EMA
GMP compliance (high burden of documentation already in early phase of application even for clinical trials driven by academia)	A GMP‐specific guideline for ATMPs including provisions for early clinical trial material is currently under development by the Commission in consultation with EMA
Product chain identity	Develop a general identifier encoding for all relevant information for the hospital and manufacturer to circumvent patient–product mismatches
Toxicities in clinical trials	Better animal models to predict the potential toxicities of CARs

In the EU, the manufacture of investigational medicinal products has to be in accordance with GMP. For CAR T cells, the need for GMP‐compliant manufacturing may constitute a specific hurdle in the timely translation to the clinic. One issue refers to the quality of the patient‐derived starting material which is highly variable per se, due to inter‐patient related differences in disease burden and pre‐treatment. Transduction efficiency, transgene expression levels, and copy numbers per cell are additional factors, which can all directly impact on efficacy and safety. A GMP process guaranteeing a product of comparable quality will therefore contribute to the safety profile of CAR T cells. To account for the specificities of ATMPs, GMP guidelines specific for ATMPs have been drafted by the European Commission followed by two targeted stakeholder consultations. It is expected that the document will provide appropriate and harmonized guidance for ATMP‐ and thus also CAR T cell‐specific manufacturing in the EU.

A harmonized approach for the regulation of clinical trials in Europe has been only partly achieved with Directive 2001/20/EC. The competent authorities of the EU member states still have slightly different requirements for clinical trial approval. For example, classification of CAR T cells as a genetically modified organism (GMO) is viewed differently in EU member states so that an environmental risk assessment is required in some but not all member states. Researchers and developers criticize this lack of harmonization, and the need for a risk assessment for each new type of CAR T cell is perceived as unnecessary burden (Table 2).

In addition, different application forms are used in different EU member states and variable approval timelines apply, which complicate the initiation of multi‐center trials involving several EU member states. To address these points as well as to breakdown the complexity of the procedures for academic researches, the current regulatory system for clinical trials has been fundamentally revised (Abou‐El‐Enein & Schneider, 2016). In April 2014, the European Parliament and Council adopted the new EU Clinical Trials Regulation (Regulation (EU) No 536/2014) in which an electronic EU portal and database will be set up by EMA, the European Council (EC), and the Member States as a single‐entry point for clinical trial applications. Until this will come into force, the voluntary harmonization procedure (VHP) supports applications for multinational clinical trials. Under this initiative, applications for multi‐center trials are evaluated in a single procedure by a joint effort of the national competent authorities of those Member States in which the trial is planned to be conducted (Renner *et al*, 2015). This does not only facilitate the application procedure but also requires regulators to exchange and harmonize their views about a specific product.

Another important hurdle refers to the lack of disseminated knowledge about clinical trial applications for ATMPs, GMP‐compliant manufacturing, non‐clinical characterization as well as clinical trial design. With the rise of CAR T cells, the pharmaceutical industry enters the field of highly individualized cell‐based gene therapy products. In fact, they cannot use established pipelines of clinical translation used for biotechnological products like blinatumomab, but must rely on academic research for pivotal knowledge [only 20% of CAR T cell trials are sponsored by pharmaceutical industry (Datasets EV1 and EV2)]. The specific scientific and regulatory requirements of ATMPs represent a challenge not only for academic developers and small biotech companies but also for the pharmaceutical industry and can be expected to result in delays in clinical investigation. Regulatory and scientific guidelines are a valuable source to get insight into the requirements for the translation of CAR T cells (Appendix Table S2). However, guidelines covering specifically the field of CAR T cells have not yet become available, indicating the difficulties of keeping pace with the rapidly evolving science in the field of ATMPs. Before initiating a clinical trial, developers are therefore encouraged to seek early on regulatory and scientific advice with competent authorities for clinical trials. For later phases of clinical development with a view on marketing authorization, seeking scientific advice from EMA is highly recommended.

As an autologous product, good distribution practice (GDP) rules are highly relevant for CAR T cells when transported from the manufacturing site to the hospital site, which can sometimes mean delivery to a different continent. Ensuring unambiguous tracking of the product to prevent patient–product mismatch is of critical importance. Yet, hospitals often use other product identifiers than the manufacturer with possible loss of information. In this respect, a unique and harmonized tracking process is being developed by the CARAT (Chimeric Antigen Receptors for Advanced Therapies) consortium funded by HORIZON 2020 (http://www.carat-horizon2020.eu/).

As for marketing, four CAR T cell products from three pharmaceutical companies (KTE‐C19, Kite Pharma; CTL019, Novartis; JCAR015 and JCAR017, Juno Therapeutics) have been granted access to EMA's Priority Medicines (PRIME) scheme (Mullard, 2017). This program was launched to support the development of medicines that target an unmet medical need, which is the case for cancer therapy with most CAR T cell products. Under PRIME, developers will have enhanced interaction and early dialogue with EMA, to optimize development plans and speed up evaluation.

Phase III trials have not been conducted for any of these CAR T cell products. As a matter of fact, conditional marketing authorization solely on the basis of single‐arm phase II studies has been granted before, for products such as blinatumomab or adcetris (CD30‐directed antibody–drug conjugate for treatment of Hodgkin lymphoma). Both products fulfilled the requirements by targeting a disease that is orphan and seriously debilitating or life‐threatening and by showing convincing efficacy with a positive benefit–risk balance. These examples show that marketing authorization can be based on intermediate end points likely to translate into clinical benefit, as for example the complete remission rate in the case of CAR T cell trials. Upon conditional approval, the developer will be obliged to increase the existing data base, complete ongoing studies, or provide data from new clinical studies to further define the benefit–risk ratio of the product. Whether for CAR T cell products it will be advisable to aim for randomized controlled studies with standard therapy cannot be judged at this point, as it depends among others on the long‐term outcome of ongoing trials.

After marketing authorization, pricing and reimbursement become of course important issues. Some initial but still limited experience with cell‐based ATMPs is available for Strimvelis, which costs 594,000 € for the complete treatment. For CAR T cell products, many analysts forecast prices of at least 300,000 $ per patient (Walker & Johnson, 2016). Given the fact that the estimated number of ADA‐SCID patients in the EU is 15 per year, it is likely that Strimvelis revenues will not repay the costs incurred during the long journey of its development. The example of Strimvelis is, however, notable in many ways. First of all, the only alternative for ADA‐SCID patients who do not have a HLA‐matched hematopoietic stem cell donor is enzyme replacement. Of note, 1‐year replacement therapy costs already half the amount of the once‐in‐a‐lifetime gene therapy treatment. Second, Italy has adopted a rather unique, conditional scheme for Strimvelis payment. In case of clearly sub‐optimal results after treatment, the manufacturer GlaxoSmithKline (GSK) will reimburse the insurance company or the healthcare system. Third, Strimvelis is expected to be trend setting and to pave the way to the market for other genetically modified cell‐based ATMPs targeting more common indications, such as thalassemia, and ultimately cancer. These products will then benefit from an economy of scale approach to manufacturing and take advantage of advancements in technology, which will likely abate costs.

## Conclusion

**Figure 6:**
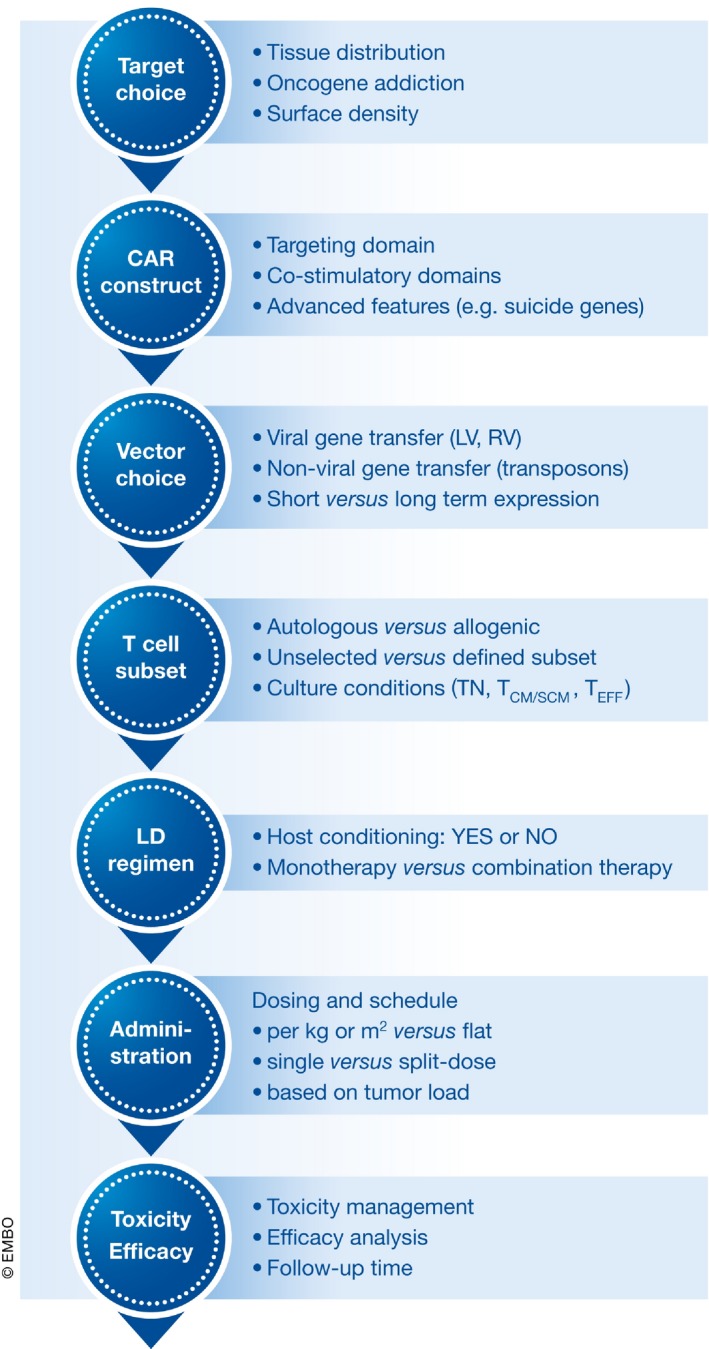
Important drivers in CAR T cell trials

CAR T cell therapy is on its way to enter clinical practice, especially for the treatment of B‐cell malignancies. The first products are likely to reach the US and European markets in the near future, according to Kite Pharma already in 2017 (DeFrancesco, 2017). Yet, with the almost infinite options in CAR specificity and design as well as in delivering, regulating, and genome editing to insert the CAR gene, it is easily conceivable that many more CAR T cell‐based products will enter into clinical research in future. This may result in better options for treating solid tumors and for simplifying the CAR T cell production process. Drawing as much information as possible from the trials completed so far will be instrumental for these new initiatives. While a detailed comparison of the different trial results is virtually impossible due to the complex nature of the CAR T cell therapy including CAR construction, the manufacturing process, the indication, and clinical trial design, some important considerations common for all types of CAR T cell therapies can be derived from the available clinical data (Fig 6).

Target choice is a critical factor, which enormously affects the subsequent study design. Parameters like its tissue distribution, tumor addiction, and surface density have to be taken into account in predicting the safety profile. For instance, expression of the target antigen on normal tissue increases the risk of on‐target/off‐tumor toxicity, whereas the lack of antigen addiction can result in its downregulation after therapy resulting in disease relapse (Lee *et al*, 2015; Turtle *et al*, 2016; Fitzgerald *et al*, 2017).

The starting material for the production of CAR T cells has so far been autologous PBMC. Allogenic CAR T cell generation and infusion after allogenic stem cell transplantation have been demonstrated to be feasible however, with low risk of graft‐versus‐host disease suggesting that CAR T cell generation may substantially reduce natural alloreactivity (Cruz *et al*, 2013; Kochenderfer *et al*, 2013; Kebriaei *et al*, 2016; Turtle *et al*, 2016). In this respect, establishment of an off‐the‐shelf CAR T cell bank can be an attractive solution, with potential to reduce time to treatment and cost. Of note, technologies like endogenous T cell receptor silencing are under current investigation (Poirot *et al*, 2015) and have recently reached the clinic for allogenic CAR T cells (Qasim *et al*, 2017). Recent clinical trial data moreover suggest that per se not only a robust manufacturing process of CAR T cells is needed, but that it is important to select for the appropriate CAR T cell phenotype and/or T‐cell composition.

As a therapy based on dividing cells, dosing of CAR T cells is a special and highly critical issue. Among the clinical trials performed so far, the dose of administered CAR T cells varied substantially. Notably, there is no correlation between the infused number of CAR T cells and the clinical outcome including related toxicities. Patients can exhibit an anti‐tumoral response even in the absence of CRS (Kebriaei *et al*, 2016; Wang *et al*, 2016). However, a high tumor burden is often associated with severe CRS and neurotoxicity (Turtle *et al*, 2016). Therefore, a risk‐adapted dosing, meaning infusion of low CAR T cell numbers for high tumor burden as well as splitting up the total dose over multiple injections (e.g., three injections each 1 day apart, accounting for 10, 30 and 60% of the total dose, respectively), may be instrumental to reduce such severe adverse events.

Along the same lines, CAR T cells can persist more than 6 years in patients and can lead to severe adverse events shortly after infusion as well as at later times. The conditioning of patients with chemotherapy or lymphodepletion regimes may also affect this. As a consequence, investigations of safety systems like suicide genes, T cell ablation, transient or controllable CAR expression are under intense evaluation (Zhang & Xu, 2017).

Finally, it must be stressed that CAR T cell therapy is still experimental and can be associated with significant risks for the patient. It becomes therefore of great importance to have a toxicity management plan in place and to identify biomarkers to predict common toxicities such as CRS. Ultimately, however, it is the overall survival data that will allow a true comparison of the long‐term benefit–risk outcome achievable with CAR T cells.

**Pending issues**

- What is the ideal CAR T cell population with regard to T‐cell subset, phenotype, and CAR construct? Direct/parallel comparison of different CAR T cell products.
- Suitable and relevant animal model to predict safety and efficacy of CAR T cell products.
- Robust and cost‐efficient manufacturing.
- How to dose CAR T cells as a dividing drug?
- Toxicity management and biomarkers.
- Defining specific regulatory requirements for CAR T cells facilitating translation to the clinic.

## Conflict of interest

The authors declare that they have no conflict of interest.

## Supporting information



Appendix

Dataset EV1

Dataset EV2

Dataset EV3

Dataset EV4

Dataset EV5
