# Supplementary material for: Clinical development of CAR T cells—challenges and opportunities in translating innovative treatment concepts
Source: EMBO Mol Med. 2017 Aug 1;9(9):1183–97. doi: 10.15252/emmm.201607485 (PMC5582407; doi:10.15252/emmm.201607485)
Supplement: Supplementary file 1 — Appendix [file EMMM-9-1183-s001.pdf]

Appendix

Clinical development of CAR-T cells – challenges and opportunities in translating innovative treatment concepts

Table of Contents:

Appendix Table S1. Long-term follow-up studies (9 total)……………………………………….p.2

Appendix Table S2 Guidelines and legislation applicable for CAR T cell therapy……………….. p.3

Appendix Table S1. Long-term follow-up studies (9 total)

Information about long-term follow-up studies as entered in ClinicalTrials.gov. Nine long-term follow-up studies for CAR T cell therapy patients were found by the end of 2016. Depicted are the study name, the targeted antigen of the CAR T cell therapy, the start date of the study, the sponsor and study sites (country) as well as the identifier.

Study	Antigen	Start	Sponso r	Country	Identifier
Long-Term Follow-Up of Recipient of Gene Transfer Research		12.2011	MDACC	USA	NCT01492036
Follow-Up Evaluation for Gene-Therapy-Related Delayed Adverse Events After Participation in Pediatric Oncology Branch Clinical Trials		11.2014	NCI	USA	NCT02315599
Longterm Follow-up of Subjects Treated With bb2121	BCMA	04.2016	bluebird	USA	NCT02786511
Long Term Follow-Up of Patients Exposed to Lentiviral-Based CD19 Directed CAR T-Cell Therapy	CD19	06.2015	Novartis	USA, Canada, Australia, EU (Spain, Austria)	NCT02445222
Long-Term Follow-up Protocol for Subjects Treated With JCAR015	CD19	08.2016	Juno	USA	NCT02813252
Long-term Follow-up Study of Patients Who Have Previously Been Exposed to UCART19	CD19	08.2016	IRIS	EU (UK)	NCT02735083
Long-term Follow-up of Subjects Exposed to Lentiviral-based CART-EGFRvIII Gene-modified Cellular Therapy Products in Cancer Studies	EGFRvIII	01.2016	ACC UPenn	USA	NCT02666248
Long-term Follow-up of Subjects Exposed to Lentiviral-based CART-meso Gene Therapy Products in Cancer Studies	Mesothelin	03.2015	ACC UPenn	USA	NCT02388828
Long-Term Follow-Up Study of Clinical Study Subjects Treated With ACTR087 Autologous T Cells Expressing Antibody-Coupled T-Cell Receptors	CD16V	07.2016	Unum	USA	NCT02840110

BCMA, B-cell maturation antigen; **EGFRvIII**, epidermal growth factor receptor subunit vIII; **ACC UPenn**, Abramson Cancer Center of the University of Pennsylvania; **bluebird**, bluebird bio; **IRIS**, Institut de Recherches Internationales Servier; **Juno**, Juno Therapeutics; **MDACC**, M.D. Anderson Cancer Center; **NCI**, National Cancer Institute; **Novartis**, Novartis Pharmaceuticals; **Unum**, Unum Therapeutics; **EU**, Europe; **UK**, United Kingdom; **USA**, United States of America

Appendix Table S2. Guidelines and legislation applicable for CAR T cell therapy.

Documents	Comment
• Human cell-based medicinal products (CBMP, 2008)<br>• Gene therapy medicinal products (GTMP, 2016)<br>• Genetically modified cells (2012)	Guidance for Advanced Therapies rather related to marketing authorization but less useful for early clinical trials
• Clinical Trials Directive 2001/20/EC<br>• Communication from the Commission CT-1<br>• National laws	General regulation for clinical trials, not ATMP specific
• Anticancer guideline EMA/CHMP 205/95/Rev4 (2012)	Guidance on all stages of clinical drug development for the treatment of malignancies, but focus on conventional drugs
• Clinical Practice Guidelines from DGHO, ESMO and ASCO	Disease and indication-specific guidance
• Good manufacturing practice (GMP) guidelines<br>(Commission Directives 91/356/EEC, 2003/94/EC, 91/412/EEC)	Guidelines are not ATMP specific, but an ATMP specific guideline is under development
