# Supplementary material for: Clinical development of CAR T cells—challenges and opportunities in translating innovative treatment concepts
Source: EMBO Mol Med. 2017 Aug 1;9(9):1183–97. doi: 10.15252/emmm.201607485 (PMC5582407; doi:10.15252/emmm.201607485)
Supplement: Supplementary file 2 — Dataset EV1 [file EMMM-9-1183-s002.doc]

## Dataset EV1. CAR gene therapy clinical trials for haematological malignancies (134 total)[**[[1]](#footnote-2)**]

| **Status** | **Antigen** | **Indication** | **Phase** | ***n*** | **Age** | **CAR** | **PT** | **SCT** | **IL2** | **Dose escalation** | **Sponsor** | **Country** | **Identifier** | **Follow-Up** | Notes / Reference |
| --- | --- | --- | --- | --- | --- | --- | --- | --- | --- | --- | --- | --- | --- | --- | --- |
| O | BCMA | MM | 1 | 38 | 18-73 years | 3rd | + | - |  | yes | NCI | USA | NCT02215967 | up to 12 month | Ref. (Ali et al, 2016) |
| O | BCMA | MM | 1 | 27 | >18 years | 2nd |  |  |  |  | ACC UPenn | USA | NCT02546167 | 2 years |  |
| O | BCMA | MM | 1 | 50 | >18 years |  | + |  |  | yes | bluebird | USA | NCT02658929 | up to 15 years  NCT02786511 |  |
| O | CD123 | AML | 1 | 30 | >18 years | 2nd | + |  |  | yes | COH | USA | NCT02159495 | Up to 15 years |  |
| O | CD123 | AML |  | 7 | >18 years | 2nd | +/- |  |  | no | UPenn | USA | NCT02623582 | 2 years |  |
| O | CD123 | Leukemia, Lymphoma | 1/2 | 45 | 4-90 years |  | + |  |  |  | SWH | China | NCT02937103 |  |  |
| O | CD138 | MM | 1/2 | 10 | 18-80 years | 2nd |  |  |  | no | CPLA | China | NCT01886976 | up to 13 years | Ref. (Guo et al, 2016) |
| O | CD16V | DLBCL, MCL, PMBCL, FL | 1 | 54 | 18-75 years |  |  |  |  |  | Unum | USA | NCT02776813 | 15 years NCT02930993 | specificity is directed by immunotherapeutic antibodies (rituximab) |
| O | CD19 | CLL | 1/2 | 30 | >18 years | 2nd | +/- |  | no | yes | MSKCC | USA | NCT00466531 | 1 year | Ref. (Brentjens et al, 2011; Brentjens et al, 2010) |
| O | CD19 | NHL | 1 | 24 | >18 years | 1st | + | - | + | yes | CHNHS | EU (UK) | NCT01493453 |  |  |
| O | CD19 | NHL, ALL, CLL | 1 | 14 |  | 2nd |  |  | no | yes | BCM | USA | NCT00586391 | up to 15 years | infusion of ipilimumab after CAR T cell therapy Ref. (Xu et al, 2014) |
| O | CD19 | DLBCL, PMBCL, MCL, DLBCL transf. FL, | 1 | 43 | 18-70 years | 2nd | + | - | +/- |  | NCI | USA | NCT00924326 | up to 1 year | Ref. (Kochenderfer et al, 2015; Kochenderfer et al, 2012; Kochenderfer et al, 2010) |
| O | CD19 | NHL, ALL, CLL | 1 | 68 |  | 2nd | no | +^allo^ | no | yes | BCM | USA | NCT00840853 | up to 15 years | Ref. (Cruz et al, 2013) |
| O | CD19 | NHL, CLL | 1 | 3 |  | 2nd | + |  | no | yes | BCM | USA | NCT00709033 | up to 15 years | Ref. (Xu et al, 2014) |
| O | CD19 | ALL | 1 | 60 | >18 years | 2nd | + |  |  | no | MSKCC | USA | NCT01044069 | 2 years | Ref. (Brentjens et al, 2011; Brentjens et al, 2013; Davila et al, 2014) |
| O | CD19 | Lymphoma | 1 | 60 | 18-75 years | 2nd | + | +/-^auto^ | +/- | yes | MDACC | USA | NCT00968760 | min 15 years  NCT01492036 | Ref. (Kebriaei et al, 2016) |
| O | CD19 | ALL, FL, CLL, MCL, DLBCL, PLL | 1 | 76 | 1-24 years | 2nd | + |  | + |  | UPenn | USA | NCT01626495 | up to 13 years | Ref. (Maude et al, 2014; Fitzgerald et al, 2016; Grupp et al, 2013) |
| O | CD19 | ALL | 1 | 12 | up to 18 years | 2nd | + | +^allo^ |  | yes | MSKCC | USA | NCT01430390 | up to 15 years |  |
| O | CD19 | DLBCL, DMBCL, DSBCL, FL, MCL | 1/2 | 57 | >18 years | 1st | + | +^auto^ |  | yes | COH | USA | NCT01318317 | min of 15 years | Ref. (Wang et al, 2016b) |
| O | CD19 | CLL | 1 | 18 | >18 years |  | + | - |  | yes | MSKCC | USA | NCT01416974 | 2 years |  |
| O | CD19 | Leukemia, Lymphoma | 1 | 140 | 1-65 years | 2nd | / | +/-^allo^ |  | yes | MDACC | USA | NCT01497184 | min 15 years  NCT01492036 | Ref. (Kebriaei et al, 2016) |
| O | CD19 | ALL, DLBCL, NHL | 1 | 53 | 1-30 years | 2nd | + | +/-^allo^ | + | yes | NCI | USA | NCT01593696 | 5 years | Ref. (Lee et al, 2015) |
| O | CD19 | ALL, NHL, SLL, CLL, BAL | 1 | 30 | 1-80 years |  | + | +/-^allo^ |  | yes | MDACC | USA | NCT02529813 | min 15 years  NCT01492036 |  |
| O | CD19 | ALL | 1 | 18 | 1-26 years |  | + | - |  | yes | SCH | USA | NCT01683279 | up to 15 years |  |
| O | CD19 | Leukemia, Lymphoma | 1 | 54 | 1-75 years |  | + | +^ucb^ |  | yes | MDACC | USA | NCT01362452 | min 15 years  NCT01492036 |  |
| O | CD19 | CLL, SLL | 2 | 61 | >18 years | 2nd |  |  |  | no | UPenn | USA | NCT01747486 | 1 year | Ref. (Fraietta et al, 2016) |
| O | CD19 | NHL | 1 | 17 | >18 years | 2nd | + | +^auto^ |  | yes | MSKCC | USA | NCT01840566 | 2 years |  |
| O | CD19 | ALL, CLL, MCL, FL, PLL | 1 | 12 | 5-90 years | 2nd |  |  |  | no | CPLA | China | NCT01864889 | up to 13 years | Ref. (Dai et al, 2015) |
| O | CD19 | CLL, NHL, ALL, DLBCL, MCL, SLL | 1/2 | 169 | >18 years | 2nd | + |  |  | yes | FHCRC | USA | NCT01865617 | min of 15 years | Ref. (Turtle et al, 2016) |
| O | CD19 | ALL | 1 | 24 | up to 26 years | 2nd | + |  |  | no | MSKCC | USA | NCT01860937 | 1 year |  |
| O | CD19 | CLL | 1 | 30 | 18-80 years |  | + |  |  | yes | MDACC | USA | NCT01653717 | min 15 years  NCT01492036 |  |
| O | CD19 | NHL | 1 | 30 | >18 years | 2nd | + | +^auto^ |  | yes | COH | USA | NCT01815749 | up to 15 years | Ref. (Wang et al, 2016b) |
| O | CD19 | ALL | 1/2 | 80 | 1-26 years | 2nd | + | +/-^allo^ |  | yes | SCH | USA | NCT02028455 | up to 10 years |  |
| O | CD19 | Lymphoma | 1/2 | 20 | >18 years | 4th |  |  |  |  | PU | China | NCT02247609 | 2 years | CAR with suicide switch |
| O | CD19 | ALL | 2 | 24 | >18 years | 2nd |  |  |  |  | UPenn | USA | NCT02030847 | 18 month |  |
| O | CD19 | NHL | 2 | 51 | >18 years |  |  |  |  | no | UPenn | USA | NCT02030834 | 15 month | murine vs. humanized CAR |
| O | CD19 | NHL, CLL, ALL | 1 | 14 | up to 75 years | 2/3 | + |  |  | yes | BCM | USA | NCT01853631 | up to 15 years |  |
| O | CD19 | NHL, MCL | 1/2 | 2 | 50-80 years | 2nd |  |  |  | yes | CPLA | China | NCT02081937 | up to 10 years |  |
| O | CD19 | ALL, DLBCL | 1 | 50 | 1-24 years | 2nd |  |  |  |  | UPenn | USA | NCT02374333 |  | humanized CAR construct;  post-cell therapy |
| O | CD19 | Lymphoma, Leukemia | 1/2 | 15 | >18 years | 3rd | + |  |  |  | UU | EU (SE) | NCT02132624 | 2 years |  |
| O | CD19 | NHL, ALL, CLL | 1 | 40 |  | 2nd | + | +^allo^ |  | yes | BCM | USA | NCT02050347 | up to 15 years |  |
| O | CD19 | DLBCL, MCL, NHL | 1 | 51 | >18 years |  | - | +^auto^ |  | yes | COH | USA | NCT02051257 | min of 15 years |  |
| O | CD19 | NHL | 1/2 | 18 | 20-70 years | 2nd | + | - |  | yes | JMU | Japan | NCT02134262 | up to 15 years |  |
| O | CD19 | MM | 1 | 13 | >18 years | 2nd | + | +^auto^ |  |  | UPenn | USA | NCT02135406 | 2 years | Ref. (Garfall et al, 2015) |
| O | CD19 | ALL | 2 | 67 | 3-21 years |  |  |  |  | no | Novartis | USA | NCT02228096 | 1 year |  |
| O | CD19 | ALL | 1 | 48 | >18 years | 2nd | + |  |  | yes | COH | USA | NCT02146924 | min 15 years | cetuximab for CAR-T cell ablation possible |
| O | CD19 | NHL, CLL, PLL | 1 | 48 | >18 years | 2nd | + | +/-^allo^ |  | yes | COH | USA | NCT02153580 | min 15 years |  |
| O | CD19 | HL |  | 16 | >18 years | 2nd |  |  |  |  | ACC UPenn | USA | NCT02277522 | 2 years |  |
| O | CD19 | ALL, CLL, NHL | 1/2 | 45 | 12-75 years |  |  |  |  | yes | SWH | China | NCT02349698 | 3 years |  |
| O | CD19 | DLBCL, transformed FL, MLBCL | 1/2 | 124 | >18 years |  | + | - |  | no | Kite | USA | NCT02348216 | 1 year |  |
| O | CD19 | CLL, ALL, Lymphoma | 1 | 36 | 1-85 years | 2nd | + |  |  |  | SSPH | China | NCT02456350 | 2 years | CAR with suicide switch |
| O | CD19 | ALL | 2 | 78 | 3-21 years |  | + |  |  | no | Novartis | USA, CA, AU, JP, EU (AT, BE, FR, DE, IT, NO, ES) | NCT02435849 | 5 years |  |
| O | CD19 | ALL, CLL, NHL |  | 30 | up to 60 years |  |  |  |  |  | ICT | China | NCT02813837 | 1 year |  |
| O | CD19 | Lymphoma, Leukemia | 1/2 | 40 | 12-70 years | 2nd |  |  |  |  | TUSM | China | NCT02537977 | 2 years |  |
| O | CD19 | DLBCL | 2 | 118 | >18 years |  |  | - |  |  | Novartis | USA ,CA, AU, JP, EU (DE, AT, FR, IT, NL, NO) | NCT02445248 | up to 15 years NCT02813252 |  |
| O | CD19 | ALL | 2 | 110 | >18 years |  | +/- |  |  | no | Juno | USA | NCT02535364 | up to 15 years  NCT02813252 |  |
| O | CD19 | Lymphoma | 1/2 | 60 | 18-80 years | 2nd | + |  |  |  | FCHG | China | NCT02547948 | 2 years |  |
| O | CD19 | B cell Leukemia | 1/2 | 20 | >18 years |  | + |  |  | yes | SMMU | China | NCT02644655 | up to 15 years |  |
| O | CD19 | ALL | 1 | 10 | >60 years |  | + |  |  |  | AHCAMM | China | NCT02799550 | 1 year |  |
| O | CD19 | DLBCL | 1/2 | 24 | 3-75 years |  |  |  |  |  | SCT | China | NCT02728882 | up to 3 years |  |
| O | CD19 | ALL | 1/2 | 24 | 3-75 years |  |  |  |  |  | SCT | China | NCT02735291 | up to 3 years |  |
| O | CD19 | HL |  | 10 | 18-24 years | 2nd | + |  |  | no | UPenn | USA | NCT02624258 |  |  |
| O | CD19 | ALL | 1/2 | 75 | >18 years |  | + |  |  | no | Kite | USA | NCT02614066 | 1 year |  |
| O | CD19 | MCL | 2 | 70 | >18 years |  | + | - |  | no | Kite | USA | NCT02601313 | 1 year |  |
| O | CD19 | ALL | 1 | 5 | 18-65 years | 3rd |  |  |  |  | AHAMM | China | NCT02186860 | 2 years |  |
| O | CD19 | DLBCL, FL, MCL | 1/2 | 20 | 18-70 years |  |  |  |  |  | XHC | China | NCT02652910 | 1 year |  |
| O | CD19 | DLBCL, FL, MCL, NHL, MLBCL | 1 | 144 | >18 years |  | + |  |  | no | Juno | USA | NCT02631044 | up to 15 years (separat trail) |  |
| O | CD19 | CLL, SLL |  | 15 | >18 years |  |  |  |  |  | UPenn | USA | NCT02640209 | 26 month | post-Ibrutinib treatment |
| O | CD19 | ALL | 1/2 | 75 | 2-21 years |  | + |  |  | no | Kite | USA | NCT02625480 | 1 year |  |
| O | CD19 | ALL, CLL, DLBCL NHL, HL | 1 | 64 | 18-73 years |  | + |  |  | yes | NCI | USA | NCT02659943 | up to 15 years | fully humanized CAR |
| O | CD19 | Leukemia | 1/2 | 30 | 1-70 years | 2nd | + |  |  | no | SGC | China | NCT02672501 |  |  |
| O | CD19 | ALL, CLL, NHL | 1 | 100 | 18-70 years |  | + |  |  | yes | BDB | China | NCT02546739 | 3 years |  |
| O | CD19 | Leukemia | 1 | 30 | 14-65 years |  |  |  |  | yes | GGH | China | NCT02822326 | 1 year |  |
| O | CD19 | DLBCL, FL, CLL, MCL, ALL | 1/2 | 20 | 5-70 years | 2nd |  |  |  | no | SAHHUT | China | NCT02685670 | 1 year |  |
| O | CD19 | ALL, BL | 1 | 18 | up to 24 years |  | + |  |  | no | UCL | EU (UK) | NCT02443831 | up to 10 years |  |
| O | CD19 | Leukemia, lymphoma | 1/2 | 50 | >10 years | 2nd |  |  |  | yes | XMU | China | NCT02782351 | 1 year | humanized CAR |
| O | CD19 | ALL | 1 | 20 | 1-60 years |  | + |  |  | no | BSBT | China | NCT02810223 | 2 years |  |
| O | CD19 | DLBCL | 1 | 12 | 16-65 years |  | + | - | no | yes | UCL | EU (UK) | NCT02431988 | 1 years |  |
| O | CD19 | MM | 2 | 25 | 18-70 years |  | + | +^auto^ |  |  | ACC UPenn | USA | NCT02794246 | 3 years |  |
| O | CD19 | Lymphoma | 1 | 20 | >18 years |  | + |  |  | yes | PU | China | NCT02842138 | 2 years |  |
| O | CD19 | ALL | 1 | 10 | 0,5-17 years |  |  |  |  |  | IRIS | EU (UK) | NCT02808442 | up to 15 years NCT02735083 |  |
| O | CD19 | ALL | 2 | 67 | >18 years |  |  |  |  | no | ACC UPenn | USA | NCT02167360 | 1 year |  |
| O | CD19 | ALL, NHL | 1/2 | 40 | 1-39 years | 2nd | + |  |  | no | SMC | Israel | NCT02772198 | 2 years |  |
| O | CD19 | ALL, CLL, FL, MCL, DLBCL, PLL | 1/2 | 10 | >14 years |  |  |  |  |  | PsersonGen | China | NCT02851589 | 2 years |  |
| O | CD19 | ALL, CLL | 1 | 12 | >16 years |  |  |  |  | yes | KCH | EU (UK) | NCT02746952 | up to 15 years NCT02735083 |  |
| O | CD19 | ALL | 1 | 20 | 4-70 years |  | + |  |  |  | HCH | China | NCT02924753 | 13 years |  |
| O | CD19 | DLBCL | 1/2 | 31 | >18 years |  | + | - |  |  | Kite | USA | NCT02926833 |  | pretreatment with atezolizumab (anti-PD-L1) |
| O | CD19 | ALL, CLL, FL, MCL, DLBCL, PLL | 1/2 | 10 | >18 years |  |  |  |  |  | PsersonGen | China | NCT02819583 | 2 years |  |
| O | CD19 | Leukemia, Lymphoma | 1 | 45 | 18-65 years | 2nd | + |  |  |  |  | China | NCT02933775 |  |  |
| O | CD19 | ALL | 2 | 24 | >18 years | 2nd |  |  |  |  | UPenn | USA | NCT02935543 |  |  |
| O | CD19 | Leukemia, Lymphoma | 1 | 12 | 16-70 years |  |  | +^allo^ |  | yes | UCL | UK | NCT02893189 | 3 years |  |
| O | CD19 | DLBCL, MLBCL | 1 | 42 | >18 years |  | + |  |  | yes | FHCRC | USA | NCT02706405 | min of 15 years | combination with Durvalumab (PD-L1) |
| O | CD19 | ALL | 1 | 20 | 16-65 years | 2nd | + |  |  | yes |  | UK | NCT02935257 | 5 years |  |
| O | CD19 | Leukemia, Lymphoma | 2 | 100 | 14-75 years |  | + |  |  |  | SWH | China | NCT02846584 | 2 years | HSCT after CAR treatment |
| C | CD19 | FL | 1 | 5 | 16-70 years |  | + |  | +/- | no | COH | USA | NCT00182650 | min of 15 years |  |
| C | CD19 | DLBCL | 1 | 2 |  | 1st | + | +^auto^ | + | yes | COH | USA | IRB 01160 |  | CAR with suicide gene  Ref. (Jensen et al, 2010) |
| C | CD19 | ALL, CLL, DLBCL, FL, MCL, PLL |  | 110 | >18 years | 2nd |  |  | + |  | ACC UPenn | USA | NCT01029366 |  | Ref. (Maude et al, 2014; Kalos et al, 2011; Porter et al, 2011; Porter et al, 2015) |
| S | CD19 | NHL, HL | 1 | 42 | 18-75 years | 2nd | + | +^allo^ | no | yes | NCI | USA | NCT01087294 | up to 15 years | Ref. (Kochenderfer et al, 2013) |
| C | CD19 | ALL | 1 | 2 | >18 years | 2nd |  | +^allo^ |  | no | UPenn | USA | NCT01551043 | up to 15 years |  |
| C | CD19 | ALL, DLBCL, MCL, CLL | 1/2 | 1 | 18-75 years |  |  | +^allo^ |  | no | FHCRC | USA | NCT01475058 | up to 15 years |  |
| T | CD19 | ALL | 1/2 | 29 | up to 18 years | 1st |  | +^allo^ |  | no | UCL | EU (UK, DE) | NCT01195480 |  |  |
| W | CD19 | ALL, NHL, SLL, CLL, FL, MCL, BAL | 1 |  | 18-70 years |  | + | +^auto^ |  | yes | MDACC | USA | NCT02274506 | min 15 years  NCT01492036 |  |
| U | CD19 | NHL |  | 6 | 46-59 years | 1/2 | + |  | no | yes | BCM | USA |  |  | Ref. (Savoldo et al, 2011) |
| O | CD19/CD20 | DLBCL | 1/2 | 40 | 14-75 years |  | + |  |  |  | SWH | China | NCT02737085 | 2 years | CD20-CAR and CD19-CAR T cells infused together |
| O | CD19/CD22 | Leukemia, Lymphoma | 1/2 | 20 | 18-70 years | 2nd |  |  |  |  | XMU | China | NCT02903810 |  | CD19 and CD22 CAR T cells infused |
| O | CD20 | ALL, CLL, PLL, DLBCL, FL, MCL | 1/2 | 50 | 18-90 years | 2nd |  |  |  |  | CPLA | China | NCT01735604 | up to 13 years | Ref. (Wang et al, 2014) |
| O | CD20 | Leukemia, Lymphoma | 1/2 | 45 | 14-75 years |  | + |  |  |  | SWH | China | NCT02710149 | 2 years |  |
| O | CD20 | Leukemia, Lymphoma | 2 | 100 | 14-75 years |  | + |  |  |  | SWH | China | NCT02846584 | 2 years | prior to HSCT |
| C | CD20 | FL | 1 | 5 |  | 1st | + | - | no | yes | COH | USA | IRB 98142 |  | Ref. (Jensen et al, 2010) |
| C | CD20 | Leukemia, Lymphoma | 1 | 12 |  | 1st | + | - | +/- |  | FHCRC | USA | NCT00012207 | up to 2 years | Ref. (Till et al, 2008) |
| C | CD20 | CLL, FL, MCL, SLL, MZL | 1 | 12 |  | 2nd | + | - | + |  | FHCRC | USA | NCT00621452 | up to 2 years | Ref. (Till et al, 2012) |
| O | CD22 | FL, ALL, NHL, DLBCL | 1 | 57 | 1-30 years |  | + |  |  | yes | NCI | USA | NCT02315612 | up to 15 years |  |
| O | CD22 | ALL | 1 | 15 | >18 years |  |  |  |  |  | UPenn | USA | NCT02588456 | 3 years |  |
| O | CD22 | ALL | 1 | 15 | 1-24 years |  | / |  |  | no | UPenn | USA | NCT02650414 | up to 15 years |  |
| O | CD22 | DLBCL, FL, MCL | 1 | 20 | 18-70 years |  | + |  |  |  | XHC | China | NCT02721407 | 6 month | resistant to prior CD19-CAR T cell therapy |
| O | CD22 | Leukemia, Lymphoma | 1/2 | 10 | 4-60 years |  |  |  |  | no | XMU | China | NCT02794961 | 1 year | humanized |
| O | CD22 | Leukemia, Lymphoma | 1/2 | 45 | 4-90 years |  | + |  |  |  | SWH | China | NCT02935153 |  |  |
| O | CD30 | NHL, HL | 1 | 18 |  |  |  |  |  | yes | BCM | USA | NCT01192464 | up to 15 years |  |
| O | CD30 | NHL, HL | 1 | 18 |  | 2nd |  |  |  | yes | UNCL | USA | NCT01316146 | up to 15 years |  |
| O | CD30 | Lymphoma | 1/2 | 20 | >18 years | 4th |  |  |  |  | PU | China, USA | NCT02274584 | 2 years | CAR with suicide switch |
| O | CD30 | NHL, HL | 1/2 | 30 | 16-80 years | 2nd | + |  |  | yes | CPLA | China | NCT02259556 | 1 year | Ref. (Wang et al, 2016a) |
| O | CD30 | Lymphoma | 1 | 18 | >3 years | 2nd |  | +^auto^ |  | yes | UNCL | USA | NCT02663297 | up to 15 years |  |
| O | CD30 | NHL, HL | 1/2 | 31 | >18 years | 2nd | + |  |  | yes | UNCL | USA | NCT02690545 | up to 15 years |  |
| O | CD30 | HL, NHL | 1 | 14 |  |  | + | +^auto^ |  | yes | BCM | USA | NCT02917083 | 15 years |  |
| O | CD30 | CD30+ cancer | 1 | 15 | 18-70 years |  |  | - |  |  | UC | EU (DE) | NCT01645293 |  |  |
| O | CD33 | AML | 1/2 | 10 | 5-90 years | 1/2 |  |  |  |  | CPLA | China | NCT01864902 | up to 13 years | Ref. (Wang et al, 2015) |
| O | CD33 | AML | 1 | 12 | >50 years |  | + |  |  |  | AHCAMM | China | NCT02799680 | 1 year |  |
| O | CD70 | CD70+ cancer | 1/2 | 113 | 18-70 years |  | + |  | + |  | NCI | USA | NCT02830724 | 5 years | humanized CAR construct |
| O | Ig k | CLL, NHL, MM | 1 | 54 | >18 years | 2nd | + |  |  | yes | BCM | USA | NCT00881920 |  | Ref. (Ramos et al, 2016) |
| O | IL-1RAP | CLL |  | 40 | 18-75 years |  |  |  |  |  | CHUB | EU (FR) | NCT02842320 | 2 years |  |
| O | Lewis Y | MM, AML, MDS | 1 | 6 | >18 years | 2nd |  |  | no |  | PMCC | Australia | NCT01716364 | Up to 3 years | Ref. (Ritchie et al, 2013) |
| O | NKG2D ligand | AML, MDS, MM | 1 | 12 | >18 years |  | no | - |  | yes | CM | USA | NCT02203825 | Up to 15 years |  |
| O | ROR1 | CLL, SLL | 1 | 48 | 18-85 years | 2nd | + |  |  | yes | MDACC | USA | NCT02194374 | Up to 15 years NCT01492036 |  |
| O | ROR1 | CLL, MCL, ALL, | 1 | 60 | >18 years |  | + |  |  | yes | FHCRC | USA | NCT02706392 | Up to 15 years |  |

**C**, completed; **O**, ongoing; **T**, terminated; **S**, suspended; **W**, withdrawn; **U**, unknown; ***n****,* number of enrolled patients; **CAR**, generation of the CAR construct; **PT**, pretreatment like lymphodepletion or chemotherapy; **SCT**, stem cell transplantation; **IL2**, systemic IL-2 administration; **+**, yes; **-**, no; **+/-**, variable; **/**, optional; **allo**, allogenic; **auto**, autologous; **ucb**, umbilical cord blood transplantation; **BCMA**, B-cell maturation antigen; **Ig k**, Ig kappa light cahin; **IL-1RAP**, Interleukin-1 receptor accessory protein; **ROR1**, receptor tyrosine kinase-like orphan receptor; **ALL**, acute lymphoid leukemia; **AML**, acute myeloid leukemia; **BAL**, Biphenotypic acute leukaemia; **BL**, burkitt lymphoma; **CLL**, chronic lymphocytic leukemia; **DLBCL**, diffuse large B cell lymphoma; **DMBCL**, diffuse medium B cell lymphoma; **DSBCL**, diffuse small B cell lymphoma; **FL**, follicular lymphoma; **HL**, Hodgkin lymphoma; **MCL**, mantle cell lymphoma; **MDS**, myelodysplastic sndrome; **MLBCL**, mediastinal large B-cell lymphoma; **MM**, multiple myeloma; **MZL**, marginal zone lymphomas; **NHL**, non Hodgkin lymphoma; **PLL**, B cell prolymphocytic leukemia; **PMBCL**, Primary mediastinal B-cell lymphoma; **SLL**, small lymphocytic lymphoma; **ACC UPenn**, Abramson Cancer Center of the University of Pennsylvania; **AHAMM**, Affiliated Hospital to Academy of Military Medical Sciences; **AHCAMM**, The Affiliated Hospital of the Chinese Academy of Military Medical Sciences; **BCM**, Baylor College of Medicine; **BDB**, Beijing Doing Biomedical; **bluebird**, bluebird bio; **BSBT**, Beijing Sanwater Biological Technology; **CHNHS**, Christie Hospital NHS Foundation Trust; **CHUB**, Centre Hospitalier Universitaire de Besancon; **CM**, Celdara Medical; **COH**, City of Hope Medical Center; **CPLA**, Chinese PLA General Hospital; **FCHG**, Fuda Cancer Hospital, Guangzhou; **FHCRC**, Fred Hutchinson Cancer Research Center; **GGH**, Guangdong General Hospital; **HCH**, Henan Cancer Hospital; **ICT**, Innovative Cellular Therapeutics; **IRIS**, Institut de Recherches Internationales Servier; **JMU**, Jichi Medical University; **Juno**, Juno Therapeutics; **KCH**, King's College Hospital NHS Trust; **Kite**, Kite Pharma; **MDACC**, M.D. Anderson Cancer Center; **MSKCC**, Memorial Sloan Kettering Cancer Center; **NCI**, National Cancer Institute; **Novartis**, Novartis Pharmaceuticals; **PMCC**, Peter MacCallum Cancer Centre, Australia; **PsersonGen**, PersonGen BioTherapeutics ; **PU**, Peking University; **SAHHUT**, The Second Affiliated Hospital of Henan University of Traditional Chinese Medicine; **SCH**, Seattle Children's Hospital; **SCT**, Sinobioway Cell Therapy; **SCT**, Sinobioway Cell Therapy ; **SGC**, Shanghai GeneChem ; **SMC**, Sheba Medical Center; **SMMU**, Second Military Medical University; **SSPH**, Shenzhen Second People's Hospital; **SWH**, Southwest Hospital, China; **TUSM**, Tongji University School of Medicine; **UC**, University of Cologne; **UCL**, University College, London; **UNCL**, UNC Lineberger Comprehensive Cancer Center; **Unum**, Unum Therapeutics; **UPenn**, University of Pennsylvania; **UU**, Uppsala University; **XHC**, Xinqiao Hospital of Chongqing; **XMU**, Xuzhou Medical University; **AT**, Austria; **AU**, Australia; **BE**, Belgium; **CA**, Canada; **DE**, Germany; **ES**, Spain; **EU**, Europe; **FR**, France; **IT**, Italy; **JP**, Japan; **NL**, Netherlands; **NO**, Norway; **SE**, Sweden; **UK**, United Kingdom; **USA**, United States of America

References

Ali SA, Shi V, Maric I, Wang M, Stroncek DF, Rose JJ, Brudno JN, Stetler-Stevenson M, Feldman SA, Hansen BG, Fellowes VS, Hakim FT, Gress RE & Kochenderfer JN (2016) T cells expressing an anti-B-cell maturation antigen chimeric antigen receptor cause remissions of multiple myeloma. *Blood* **128:** 1688–1700

Brentjens R, Yeh R, Bernal Y, Riviere I & Sadelain M (2010) Treatment of chronic lymphocytic leukemia with genetically targeted autologous T cells: case report of an unforeseen adverse event in a phase I clinical trial. *Molecular therapy : the journal of the American Society of Gene Therapy* **18:** 666–668

Brentjens RJ, Davila ML, Riviere I, Park J, Wang X, Cowell LG, Bartido S, Stefanski J, Taylor C, Olszewska M, Borquez-Ojeda O, Qu J, Wasielewska T, He Q, Bernal Y, Rijo IV, Hedvat C, Kobos R, Curran K & Steinherz P et al (2013) CD19-targeted T cells rapidly induce molecular remissions in adults with chemotherapy-refractory acute lymphoblastic leukemia. *Science translational medicine* **5:** 177ra38

Brentjens RJ, Riviere I, Park JH, Davila ML, Wang X, Stefanski J, Taylor C, Yeh R, Bartido S, Borquez-Ojeda O, Olszewska M, Bernal Y, Pegram H, Przybylowski M, Hollyman D, Usachenko Y, Pirraglia D, Hosey J, Santos E & Halton E et al (2011) Safety and persistence of adoptively transferred autologous CD19-targeted T cells in patients with relapsed or chemotherapy refractory B-cell leukemias. *Blood* **118:** 4817–4828

Cruz CRY, Micklethwaite KP, Savoldo B, Ramos CA, Lam S, Ku S, Diouf O, Liu E, Barrett AJ, Ito S, Shpall EJ, Krance RA, Kamble RT, Carrum G, Hosing CM, Gee AP, Mei Z, Grilley BJ, Heslop HE & Rooney CM et al (2013) Infusion of donor-derived CD19-redirected virus-specific T cells for B-cell malignancies relapsed after allogeneic stem cell transplant: a phase 1 study. *Blood* **122:** 2965–2973

Dai H, Zhang W, Li X, Han Q, Guo Y, Zhang Y, Wang Y, Wang C, Shi F, Zhang Y, Chen M, Feng K, Wang Q, Zhu H, Fu X, Li S & Han W (2015) Tolerance and efficacy of autologous or donor-derived T cells expressing CD19 chimeric antigen receptors in adult B-ALL with extramedullary leukemia. *Oncoimmunology* **4:** e1027469

Davila ML, Riviere I, Wang X, Bartido S, Park J, Curran K, Chung SS, Stefanski J, Borquez-Ojeda O, Olszewska M, Qu J, Wasielewska T, He Q, Fink M, Shinglot H, Youssif M, Satter M, Wang Y, Hosey J & Quintanilla H et al (2014) Efficacy and toxicity management of 19-28z CAR T cell therapy in B cell acute lymphoblastic leukemia. *Science translational medicine* **6:** 224ra25

Fitzgerald JC, Weiss SL, Maude SL, Barrett DM, Lacey SF, Melenhorst JJ, Shaw P, Berg RA, June CH, Porter DL, Frey NV, Grupp SA & Teachey DT (2016) Cytokine Release Syndrome After Chimeric Antigen Receptor T Cell Therapy for Acute Lymphoblastic Leukemia. *Critical care medicine*

Fraietta JA, Beckwith KA, Patel PR, Ruella M, Zheng Z, Barrett DM, Lacey SF, Melenhorst JJ, McGettigan SE, Cook DR, Zhang C, Xu J, Do P, Hulitt J, Kudchodkar SB, Cogdill AP, Gill S, Porter DL, Woyach JA & Long M et al (2016) Ibrutinib enhances chimeric antigen receptor T-cell engraftment and efficacy in leukemia. *Blood* **127:** 1117–1127

Garfall AL, Maus MV, Hwang W-T, Lacey SF, Mahnke YD, Melenhorst JJ, Zheng Z, Vogl DT, Cohen AD, Weiss BM, Dengel K, Kerr NDS, Bagg A, Levine BL, June CH & Stadtmauer EA (2015) Chimeric Antigen Receptor T Cells against CD19 for Multiple Myeloma. *The New England journal of medicine* **373:** 1040–1047

Grupp SA, Kalos M, Barrett D, Aplenc R, Porter DL, Rheingold SR, Teachey DT, Chew A, Hauck B, Wright JF, Milone MC, Levine BL & June CH (2013) Chimeric antigen receptor-modified T cells for acute lymphoid leukemia. *The New England journal of medicine* **368:** 1509–1518

Guo B, Chen M, Han Q, Hui F, Dai H, Zhang W, Zhang Y, Wang Y, Zhu H & Han W (2016) CD138-directed adoptive immunotherapy of chimeric antigen receptor (CAR)-modified T cells for multiple myeloma. *Journal of Cellular Immunotherapy* **2:** 28–35

Jensen MC, Popplewell L, Cooper LJ, DiGiusto D, Kalos M, Ostberg JR & Forman SJ (2010) Antitransgene rejection responses contribute to attenuated persistence of adoptively transferred CD20/CD19-specific chimeric antigen receptor redirected T cells in humans. *Biology of blood and marrow transplantation : journal of the American Society for Blood and Marrow Transplantation* **16:** 1245–1256

Kalos M, Levine BL, Porter DL, Katz S, Grupp SA, Bagg A & June CH (2011) T cells with chimeric antigen receptors have potent antitumor effects and can establish memory in patients with advanced leukemia. *Science translational medicine* **3:** 95ra73

Kebriaei P, Singh H, Huls MH, Figliola MJ, Bassett R, Olivares S, Jena B, Dawson MJ, Kumaresan PR, Su S, Maiti S, Dai J, Moriarity B, Forget M-A, Senyukov V, Orozco A, Liu T, McCarty J, Jackson RN & Moyes JS et al (2016) Phase I trials using Sleeping Beauty to generate CD19-specific CAR T cells. *The Journal of clinical investigation* **126:** 3363–3376

Kochenderfer JN, Dudley ME, Carpenter RO, Kassim SH, Rose JJ, Telford WG, Hakim FT, Halverson DC, Fowler DH, Hardy NM, Mato AR, Hickstein DD, Gea-Banacloche JC, Pavletic SZ, Sportes C, Maric I, Feldman SA, Hansen BG, Wilder JS & Blacklock-Schuver B et al (2013) Donor-derived CD19-targeted T cells cause regression of malignancy persisting after allogeneic hematopoietic stem cell transplantation. *Blood* **122:** 4129–4139

Kochenderfer JN, Dudley ME, Feldman SA, Wilson WH, Spaner DE, Maric I, Stetler-Stevenson M, Phan GQ, Hughes MS, Sherry RM, Yang JC, Kammula US, Devillier L, Carpenter R, Nathan D-AN, Morgan RA, Laurencot C & Rosenberg SA (2012) B-cell depletion and remissions of malignancy along with cytokine-associated toxicity in a clinical trial of anti-CD19 chimeric-antigen-receptor-transduced T cells. *Blood* **119:** 2709–2720

Kochenderfer JN, Dudley ME, Kassim SH, Somerville RPT, Carpenter RO, Stetler-Stevenson M, Yang JC, Phan GQ, Hughes MS, Sherry RM, Raffeld M, Feldman S, Lu L, Li YF, Ngo LT, Goy A, Feldman T, Spaner DE, Wang ML & Chen CC et al (2015) Chemotherapy-refractory diffuse large B-cell lymphoma and indolent B-cell malignancies can be effectively treated with autologous T cells expressing an anti-CD19 chimeric antigen receptor. *Journal of clinical oncology : official journal of the American Society of Clinical Oncology* **33:** 540–549

Kochenderfer JN, Wilson WH, Janik JE, Dudley ME, Stetler-Stevenson M, Feldman SA, Maric I, Raffeld M, Nathan D-AN, Lanier BJ, Morgan RA & Rosenberg SA (2010) Eradication of B-lineage cells and regression of lymphoma in a patient treated with autologous T cells genetically engineered to recognize CD19. *Blood* **116:** 4099–4102

Lee DW, Kochenderfer JN, Stetler-Stevenson M, Cui YK, Delbrook C, Feldman SA, Fry TJ, Orentas R, Sabatino M, Shah NN, Steinberg SM, Stroncek D, Tschernia N, Yuan C, Zhang H, Zhang L, Rosenberg SA, Wayne AS & Mackall CL (2015) T cells expressing CD19 chimeric antigen receptors for acute lymphoblastic leukaemia in children and young adults: A phase 1 dose-escalation trial. *The Lancet* **385:** 517–528

Maude SL, Frey N, Shaw PA, Aplenc R, Barrett DM, Bunin NJ, Chew A, Gonzalez VE, Zheng Z, Lacey SF, Mahnke YD, Melenhorst JJ, Rheingold SR, Shen A, Teachey DT, Levine BL, June CH, Porter DL & Grupp SA (2014) Chimeric antigen receptor T cells for sustained remissions in leukemia. *N. Engl. J. Med* **371:** 1507–1517

Porter DL, Hwang W-T, Frey NV, Lacey SF, Shaw PA, Loren AW, Bagg A, Marcucci KT, Shen A, Gonzalez V, Ambrose D, Grupp SA, Chew A, Zheng Z, Milone MC, Levine BL, Melenhorst JJ & June CH (2015) Chimeric antigen receptor T cells persist and induce sustained remissions in relapsed refractory chronic lymphocytic leukemia. *Science translational medicine* **7:** 303ra139

Porter DL, Levine BL, Kalos M, Bagg A & June CH (2011) Chimeric antigen receptor-modified T cells in chronic lymphoid leukemia. *The New England journal of medicine* **365:** 725–733

Ramos CA, Savoldo B, Torrano V, Ballard B, Zhang H, Dakhova O, Liu E, Carrum G, Kamble RT, Gee AP, Mei Z, Wu M-F, Liu H, Grilley B, Rooney CM, Brenner MK, Heslop HE & Dotti G (2016) Clinical responses with T lymphocytes targeting malignancy-associated kappa light chains. *The Journal of clinical investigation*

Ritchie DS, Neeson PJ, Khot A, Peinert S, Tai T, Tainton K, Chen K, Shin M, Wall DM, Honemann D, Gambell P, Westerman DA, Haurat J, Westwood JA, Scott AM, Kravets L, Dickinson M, Trapani JA, Smyth MJ & Darcy PK et al (2013) Persistence and efficacy of second generation CAR T cell against the LeY antigen in acute myeloid leukemia. *Molecular therapy : the journal of the American Society of Gene Therapy* **21:** 2122–2129

Savoldo B, Ramos CA, Liu E, Mims MP, Keating MJ, Carrum G, Kamble RT, Bollard CM, Gee AP, Mei Z, Liu H, Grilley B, Rooney CM, Heslop HE, Brenner MK & Dotti G (2011) CD28 costimulation improves expansion and persistence of chimeric antigen receptor-modified T cells in lymphoma patients. *The Journal of clinical investigation* **121:** 1822–1826

Till BG, Jensen MC, Wang J, Chen EY, Wood BL, Greisman HA, Qian X, James SE, Raubitschek A, Forman SJ, Gopal AK, Pagel JM, Lindgren CG, Greenberg PD, Riddell SR & Press OW (2008) Adoptive immunotherapy for indolent non-Hodgkin lymphoma and mantle cell lymphoma using genetically modified autologous CD20-specific T cells. *Blood* **112:** 2261–2271

Till BG, Jensen MC, Wang J, Qian X, Gopal AK, Maloney DG, Lindgren CG, Lin Y, Pagel JM, Budde LE, Raubitschek A, Forman SJ, Greenberg PD, Riddell SR & Press OW (2012) CD20-specific adoptive immunotherapy for lymphoma using a chimeric antigen receptor with both CD28 and 4-1BB domains: pilot clinical trial results. *Blood* **119:** 3940–3950

Turtle CJ, Hanafi L-A, Berger C, Gooley TA, Cherian S, Hudecek M, Sommermeyer D, Melville K, Pender B, Budiarto TM, Robinson E, Steevens NN, Chaney C, Soma L, Chen X, Yeung C, Wood B, Li D, Cao J & Heimfeld S et al (2016) CD19 CAR-T cells of defined CD4+:CD8+ composition in adult B cell ALL patients. *The Journal of clinical investigation* **126:** 2123–2138

Wang C, Wu Z, Wang Y, Guo Y, Dai H, Wang X-H, Li X, Zhang Y-j, Zhang W-y, Chen M-x, Zhang Y, Feng K-c, Liu Y, Li S-X, Yang Q-M & Han W (2016a) Autologous T cells expressing CD30 chimeric antigen receptors for relapsed or refractory Hodgkin’s lymphoma: an open-label phase I trial. *Clinical cancer research : an official journal of the American Association for Cancer Research*

Wang Q-s, Wang Y, Lv H-y, Han Q-w, Fan H, Guo B, Wang L-l & Han W-d (2015) Treatment of CD33-directed chimeric antigen receptor-modified T cells in one patient with relapsed and refractory acute myeloid leukemia. *Molecular therapy : the journal of the American Society of Gene Therapy* **23:** 184–191

Wang X, Popplewell LL, Wagner JR, Naranjo A, Blanchard MS, Mott MR, Norris AP, Wong CW, Urak RZ, Chang W-C, Khaled SK, Siddiqi T, Budde LE, Xu J, Chang B, Gidwaney N, Thomas SH, Cooper LJN, Riddell SR & Brown CE et al (2016b) Phase 1 studies of central memory-derived CD19 CAR T-cell therapy following autologous HSCT in patients with B-cell NHL. *Blood* **127:** 2980–2990

Wang Y, Zhang W-y, Han Q-w, Liu Y, Dai H-r, Guo Y-l, Bo J, Fan H, Zhang Y, Zhang Y-j, Chen M-x, Feng K-c, Wang Q-s, Fu X-b & Han W-d (2014) Effective response and delayed toxicities of refractory advanced diffuse large B-cell lymphoma treated by CD20-directed chimeric antigen receptor-modified T cells. *Clinical immunology (Orlando, Fla.)* **155:** 160–175

Xu Y, Zhang M, Ramos CA, Durett A, Liu E, Dakhova O, Liu H, Creighton CJ, Gee AP, Heslop HE, Rooney CM, Savoldo B & Dotti G (2014) Closely related T-memory stem cells correlate with in vivo expansion of CAR.CD19-T cells and are preserved by IL-7 and IL-15. *Blood* **123:** 3750–3759

1. [?] Information about CAR T cell clinical trials collected from ClinicalTrials.gov or literature. 134 CAR T cell clinical trials for haematological malignancies were registered by the end of 2016. Depicted are the status of each trial (ongoing, completed; terminated; suspended; withdrawn; unknown), the targeted antigen, the treated indication, the phase of the trial (phase I, phase I/II, Phase II), the number and age of enrolled patients, the generation of the CAR constructed used, whether a pre-treatment like lymphodepletion or chemotherapy, stem cell transplantation, systemic IL-2 administration or a dose escalation regimen were applied, the sponsor of the trial, the country of the trial site/s, the identifier and follow-up time. [↑](#footnote-ref-2)
