# Supplementary material for: Clinical development of CAR T cells—challenges and opportunities in translating innovative treatment concepts
Source: EMBO Mol Med. 2017 Aug 1;9(9):1183–97. doi: 10.15252/emmm.201607485 (PMC5582407; doi:10.15252/emmm.201607485)
Supplement: Supplementary file 3 — Dataset EV2 [file EMMM-9-1183-s003.doc]

## Dataset EV2. CAR gene therapy clinical trials for solid tumors (78 total)[**[[1]](#footnote-2)**]

| **Status** | **Antigen** | **Indication** | **Phase** | ***n*** | **Age** | **CAR** | **PT** | **IL2** | **Dose escalation** | **Sponsor** | **Country** | **Identifier** | **Follow-Up** | Notes / Reference |
| --- | --- | --- | --- | --- | --- | --- | --- | --- | --- | --- | --- | --- | --- | --- |
| T | CAIX | RCC | 1/2 | 12 | 46-74 years | 1st | no | no | yes | EUMC | EU (NL) | DDHK97-29/P00.0040C |  | Ref. (Lamers et al, 2013; Lamers et al, 2011; Lamers et al, 2007; Lamers et al, 2006) |
| O | CEA | Various | 1 | 75 | 18-80 years |  |  |  | yes | SWH | China | NCT02349724 | up to 3 years |  |
| O | CEA | Liver Metastases | 1 | 8 | >18 years |  |  | + | no | RWMC | USA | NCT02416466 |  | hepatic artery infusion |
| O | CEA | Liver Metastases | 1/2 | 20 | 18-69 years | 2nd |  |  |  | XH | China | NCT02862704 |  |  |
| O | CEA | Liver cancer | 1 | 5 | >18 years |  |  | + | no | RWMC | USA | NCT02850536 |  | hepatic artery infusion |
| C | CEA | Adenocarcinoma | 1 |  | >18 years |  |  |  | yes | RWMC | USA | NCT00004178 | 2 month |  |
| W | CEA | Gatric Cancer | 1 |  | >18 years | 2nd |  |  | NA | RWMC | USA | NCT00429078 |  |  |
| T | CEA | Various | 1 | 14 | >18 years | 1st | + | + | yes | CRUK | EU (UK) | NCT01212887 |  |  |
| T | CEA | Colorectal cancer | 1 | 1 | >18 years | 2nd |  |  | yes | RWMC | USA | NCT00673322 | 1 month |  |
| S | CEA | Breast cancer | 1 | 26 | >18 years | 2nd |  | +/- | yes | RWMC | USA | NCT00673829 | 1 month |  |
| C | CEA | Liver metastases | 1 | 8 | >18 years | 2nd |  |  | yes | RWMC | USA | NCT01373047 | 1 month | hepatic artery infusion Ref. (Katz et al, 2015; Saied et al, 2014) |
| S | CEA | adenocarcinomas | 2 | 48 | 18-80 years | 2nd |  | + | NA | RWMC | USA | NCT01723306 | 2 years |  |
| O | c-MET | Breast cancer | 1 | 15 |  |  |  |  |  | ACC UPenn | USA | NCT01837602 | 2 years | intratumoral injection |
| O | EGFR | EGFR+ solid tumors | 1/2 | 60 | 18-80 years | 2nd |  |  |  | CPLA | China | NCT01869166 | up to 13 years | Ref. (Feng et al, 2016) |
| O | EGFR | GBM, glioma | 1 | 10 | 18-70 years |  | + | + |  | RJH | China | NCT02331693 | 2 years |  |
| O | EGFR | advanced malignancies | 1/2 | 20 | 18-65 years |  |  |  |  | NCH | China | NCT02873390 |  | CAR T cells expressing PD-1 antibody |
| O | EGFR | various | 1/2 | 20 | 18-65 years |  |  |  |  | SIMC | China | NCT02862028 |  | CAR T cells expressing PD-1 antibody |
| O | EGFRvIII | Glioma |  | 12 | >18 years |  |  |  |  | UPenn | USA | NCT02209376 | up to 15 years NCT02666248 |  |
| O | EGFRvIII | GBM | 1 | 20 | 18-70 years |  | + |  | no | BSBH | China | NCT02844062 | 2 years |  |
| O | EGFRvIII | Glioblastoma | 1 | 48 | 18-80 years |  | + |  | yes | Duke | USA | NCT02664363 | up to 18 month |  |
| S | EGFRvIII | Malignant Gliomas | 1/2 | 18 | 18-70 years | 3rd | + | + | yes | NCI | USA | NCT01454596 | 7 years |  |
| O | EpCam | Liver Cancer | 1/2 | 25 | up to 75 years |  | + |  |  | SCT | China | NCT02729493 | 35 month |  |
| O | EpCam | Stomach Cancer | 1/2 | 19 | up to 75 years |  | + |  |  | SCT | China | NCT02725125 | 35 month |  |
| O | EpCAM | Breast Cancer | 1 | 30 | 18-65 years |  | + |  |  | SU | China | NCT02915445 | 15 years |  |
| O | EphA2 | Malignt Glioma | 1/2 | 60 | 18-80 years |  |  |  |  | FCHG | China | NCT02575261 |  |  |
| O | ErbB2/Her2 | HER2+ maligncy | 1 | 19 | >3 years |  |  |  | yes | BCM | USA | NCT00889954 | up to 15 years |  |
| O | ErbB2/Her2 | Sarcoma | 1 | 36 |  | 2nd | + |  | yes | BCM | USA | NCT00902044 | up to 15 years | Ref. (Ahmed et al, 2015) |
| O | ErbB2/Her2 | GBM | 1 | 16 |  | 2nd |  |  | yes | BCM | USA | NCT01109095 | up to 15 years |  |
| O | ErbB2/Her2 | Her2+ solid tumors | 1/2 | 10 | 18-80 years | 1/2 |  |  |  | CPLA | China | NCT01935843 | up to 13 years |  |
| O | ErbB2/Her2 | Head and Neck cancer | 1 | 30 | >18 years | 2nd | - |  | yes | KCL | EU (UK) | NCT01818323 |  | intratumoral injection; ErbB ligand T1E as targeting domain; chimeric cytokine receptor (4ab) expression on CAR T cells / Ref. (van Schalkwyk et al, 2013) |
| O | ErbB2/Her2 | Breast cancer | 1/2 | 60 | 18-80 years | 2nd | + |  |  | FCHG | China | NCT02547961 | 2 years |  |
| O | ErbB2/Her2 | Glioblastoma | 1 | 14 | >18 years |  |  |  | yes | BCM | USA | NCT02442297 | up to 15 years |  |
| O | ErbB2/Her2 | Various | 1/2 | 60 | 18-80 years |  |  |  | yes | SWH | China | NCT02713984 | 3 years |  |
| T | ErbB2/Her2 | Metastatic Cancer | 1/2 | 1 | >18 years | 3rd | + | + | yes | NIHCC | USA | NCT00924287 |  | Ref. (Morgan et al, 2010) |
| O | FAP | Mesothelioma | 1 | 6 | 18-75 years | 2nd |  |  | no | ZU | EU (CH) | NCT01722149 |  | pleural effusion  Ref. (Petrausch et al, 2012) |
| O | FR-a | Ovarian cancer | 1 | 15 | >18 years | 2nd | + | no | yes | UPenn | USA |  |  | Administration of autologous untransduced PBMCs to shorten duration of lymphopenia  Ref. (Kandalaft et al, 2012) |
| C | FR-a | Ovarian cancer | 1 |  | >18 years | 1st | no | +/- | yes | NCI | USA | NCT00019136 |  | Ref. (Kershaw et al, 2006) |
| O | GD2 | Neuroblastoma | 1 | 19 | up to 21 years | 1st | + |  | yes | BCM | USA | NCT00085930 | up to 15 years | Ref. (Louis et al, 2011; Pule et al, 2008) |
| O | GD2 | Neuroblastoma | 1 | 11 |  | 3rd | +/- |  | yes | BCM | USA | NCT01822652 | up to 15 years | CAR with suicide switch, combination with pembrolizumab (anti-PD-1) |
| O | GD2 | Various | 1 | 72 | 1-35 years | 3rd | + |  | yes | NCI | USA | NCT02107963 | up to 3 years | CAR with suicide switch |
| O | GD2 | Sarcomas | 1 | 26 |  | 3rd |  |  | yes | BCM | USA | NCT01953900 | up to 15 years | CAR with suicide switch |
| O | GD2 | Neuroblastoma | 1 | 27 | >1 years | 2nd | + |  | no | CRUK | EU (UK) | NCT02761915 | up to 15 years |  |
| O | GD2 | Neuroblastoma | 2 | 30 | 1-14 years | 4th | + |  |  | ZH | China | NCT02765243 | 1 year |  |
| O | GD2 | Neuroblastoma | 1/2 | 22 | 1-14 years |  | + |  | yes | SCT | China | NCT02919046 | 3 years |  |
| C | GD2 | Neuroblastoma | 1 | 5 | 1,5-17 years |  |  |  | NA | CMHKC | USA | NCT01460901 | up to 15 years |  |
| O | GPC3 | HCC | 1 | 20 | 18-70 years |  |  |  |  | RJH | China | NCT02395250 |  |  |
| O | GPC3 | HCC | 1/2 | 60 | 18-70 years |  |  |  |  | FCHG | China | NCT02723942 | 3 month |  |
| O | GPC3 | HCC | 1/2 | 30 | 18-69 years | 2nd | + |  |  | SGC | China | NCT02715362 |  | transcatheter arterial infusion |
| O | GPC3 | LSCC | 1 | 20 | 18-70 years |  | + |  | yes | Carsgen | China | NCT02876978 |  | comparison of 1st and 2nd CAR |
| O | GPC3 | Hepatocellular Carcinoma | 1 | 14 | >18 years |  | + |  | yes | BCM | USA | NCT02905188 | 15 years |  |
| O | GPC3 | GPC3+ solid tumor | 1 | 14 | 1-18 years |  | + |  | yes | BCM | USA | NCT02932956 | 15 years |  |
| O | IL-13Ra2 | Malignt Glioma | 1 | 75 | 18-75 years | 2nd |  |  | yes | COH | USA | NCT02208362 | up to 15 years | intracranial administration |
| C | IL-13Ra2 | brain and CNS tumors | 1 | 3 | 18-70 years | 1st |  |  | yes | COH | USA | NCT00730613 | min 15 years | intracranial injection; IL-13 as binding domain Ref. (Brown et al, 2015) |
| C | IL-13Ra2 | Malignant Glioma | 1 | 6 | 18-70 years | 1st |  | + | NA | COH | USA | NCT01082926 |  | intratumoral injection |
| O | L1-CAM | Neuroblastoma | 1 | 80 | up to 18 years | 2/3 | + |  | yes | SCH | USA | NCT02311621 | up to 15 years |  |
| C | L1-CAM | Neuroblastoma | 1 | 6 | 1-17 years | 1st | + | +/- | yes | FHCRC | USA | NCT00006480 |  | ganciclovir to ablate CAR T cells / Ref. (Park et al, 2007) |
| O | Mesothelin | MPM | 1 | 18 | >18 years | 2nd |  |  | no | ACC UPenn | USA | NCT01355965 |  | T cells with transiently expressed CAR / Ref. (Beatty et al, 2014; Maus et al, 2013) |
| O | Mesothelin | MPDAC | 1 | 16 | >18 years | 2nd |  |  | no | ACC UPenn | USA | NCT01897415 |  |  |
| O | Mesothelin | various | 1 | 19 | >18 years | 2nd | +/- |  | yes | UPenn | USA | NCT02159716 | up to 15 years NCT02388828 |  |
| O | Mesothelin | Malignt Pleural Disease | 1 | 24 | >18 years | 2nd | +/- |  | yes | MSKCC | USA | NCT02414269 | 1 years | pleural catheter infusion |
| O | Mesothelin | Pancreatic | 1 | 12 | >18 years | 2nd | + |  | yes | UPenn | USA | NCT02465983 | up to 15 years NCT02388828 | Combined with anti-CD19 (humanized) CAR T cells |
| O | Mesothelin | Various | 1 | 20 | 18-70 years | 2nd |  |  | yes | CPLA | China | NCT02580747 | 1 years |  |
| O | Mesothelin | Pancreatic Cancer | 1 | 30 | 18-69 years | 2nd | + |  | no | SGC | China | NCT02706782 |  |  |
| O | Mesothelin | Breast Cancer | 1 | 24 | >18 years |  | + |  | yes | MSKCC | USA | NCT02792114 | 2 years |  |
| O | Mesothelin | mesothelin+ tumors | 1 | 20 | 18-70 years |  | + |  | yes | CMGH | China | NCT02930993 |  |  |
| S | Mesothelin | various | 1/2 | 15 | 18-70 years |  | + | + | yes | NCI | USA | NCT01583686 | 6 years |  |
| O | MUC1 | HCC, NSCLC, TNBC, PC | 1/2 | 20 | 18-70 years | 3rd |  |  |  | PsersonGen | China | NCT02587689 | 2 years | intratumoral injection  Ref. (You et al, 2016) |
| O | MUC1 | Malignt Glioma,  CC, GC | 1/2 | 20 | 18-80 years |  |  |  |  | PsersonGen | China | NCT02617134 | 2 years |  |
| O | MUC16ecto | various | 1 | 30 | >18 years | 2nd | +/- |  | yes | MSKCC | USA | NCT02498912 |  | secretion of IL-12 by CAR T cells; intraperitoneal catheter insertion |
| O | MUC16ecto | Ovarian cancer | 1 |  |  | 2nd | +/- | no | yes | MSKCC | USA |  |  | intraperitoneally injection; IL-12 secretion by CAR T cells / Ref. (Koneru et al, 2015) |
| O | PD-L1 | GBM | 1 | 20 | 18-70 years |  | + |  | yes | BSBH | China | NCT02937844 |  | extracellular domain of PD1 used as ligand (scFv) |
| O | PSCA | Pancreatic Cancer | 1 | 30 | >18 years |  |  |  | yes | Bellicum | USA | NCT02744287 |  |  |
| O | PSMA | Prostate cancer | 1 | 18 | >18 years |  | + |  | yes | MSKCC | USA | NCT01140373 | 5 years |  |
| S | PSMA | Prostate cancer | 1/2 | 12 | 18-80 years |  | + | + | yes | RWMC | USA | NCT01929239 |  |  |
| S | PSMA | Prostate cancer | 1 | 18 | >18 years | 1st | + | + | yes | RWMC | USA | NCT00664196 | 1 month |  |
| T | PSMA | Prostate Cancer | 1 | 6 | 51-75 years | 1st | + | + | yes | RWMC | USA | BB-IND 12084 |  | Ref. (Junghans et al, 2016) |
| O | ROR1 | NSCLC,  Breast cancer (TNBC) | 1 | 60 | >18 years |  | + |  | yes | FHCRC | USA | NCT02706392 | Up to 15 years |  |
| C | VEGFR-2 | various | 1/2 | 24 | 18-70 years |  | + | + | yes | NIHCC | USA | NCT01218867 | 6 years |  |

**C**, completed; **O**, ongoing; **T**, terminated; **S**, suspended; **W**, withdrawn; ***n****,* number of enrolled patients; **CAR**, generation of the CAR construct; **PT**, pretreatment like lymphodepletion or chemotherapy; **SCT**, stem cell transplantation; **IL2**, systemic IL-2 administration; **+**, yes; **-**, no; **+/-**, variable; **CAIX**, carbonic anhydrase IX; **CEA**, carcinoembryonic antigen; **c-MET**, hepatocyte growth factor receptor; **EGFR**, epidermal growth factor receptor; **EGFRvIII**, epidermal growth factor receptor subunit vIII; **EpCam**, epithelial cell adhesion molecule; **ErbB2/Her2**, human epidermal growth factor receptor 2; **EphA2**, ephrin type-A receptor 2; **FAP**, Fibroblast activation protein; **FR-a**, Folate receptor alpha; **GC**, gastric carcinoma; **GPC3**, glypican-3; **IL-13Ra2**, Interleukin-13 receptor subunit alpha-2; **MUC1**, Mucin 1; **PD-L1**, Programmed death-ligand 1; **PSCA**, prostate stem cell antigen; **PSMA**, prostate specific membrane antigen; **ROR1**, receptor tyrosine kinase-like orphan receptor; **VEGFR-2**, Vascular endothelial growth factor receptor 2; **CC**, colorectal carcinoma; **GBM**, glioblastoma multiforme; **HCC**, hepatocellular carcinoma; **LSCC**, lung squamous cell carcinoma; **MPDAC**, metastatic pancreatic ductal adenocarcinoma; **MPM**, malignant pleural mesothelioma; **NSCLC**, non-small cell lung cancer; **PC**, pancreatic carcinoma; **RCC**, renal cell carcinoma; **TNBC**, triple negative breast cancer; **ACC UPenn**, Abramson Cancer Center of the University of Pennsylvania; **BCM**, Baylor College of Medicine; **Bellicum**, Bellicum Pharmaceuticals; **BSBH**, Beijing Sanbo Brain Hospital; **Carsgen**, Carsgen Therapeutics; **CMGH**, China Meitan General Hospital; **CMHKC**, Children's Mercy Hospital Kansas City; **COH**, City of Hope Medical Center; **CPLA**, Chinese PLA General Hospital; **CRUK**, Cancer Research UK; **Duke**, Duke University; **EUMC**, Erasmus University Medical Center; **FCHG**, Fuda Cancer Hospital, Guangzhou; **FHCRC**, Fred Hutchinson Cancer Research Center; **KCL**, King's College London; **MSKCC**, Memorial Sloan Kettering Cancer Center; **NCH**, Ningbo Cancer Hospital; **NCI**, National Cancer Institute; **NIHCC**, National Institutes of Health Clinical Center (CC); **PsersonGen**, PersonGen BioTherapeutics ; **RJH**, RenJi Hospital; **RWMC**, Roger Williams Medical Center; **SCH**, Seattle Children's Hospital; **SCT**, Sinobioway Cell Therapy; **SCT**, Sinobioway Cell Therapy; **SGC**, Shanghai GeneChem ; **SIMC**, Shanghai International Medical Center; **SU**, Sichuan University; **SWH**, Southwest Hospital, China; **UPenn**, University of Pennsylvania; **XH**, Xijing Hospital; **ZH**, Zhujiang Hospital; **ZU**, University of Zurich; **CH**, Switzerland; **EU**, Europe; **NL**, Netherlands; **NO**, Norway; **UK**, United Kingdom; **USA**, United States of America

References

Ahmed N, Brawley VS, Hegde M, Robertson C, Ghazi A, Gerken C, Liu E, Dakhova O, Ashoori A, Corder A, Gray T, Wu M-F, Liu H, Hicks J, Rainusso N, Dotti G, Mei Z, Grilley B, Gee A & Rooney CM et al (2015) Human Epidermal Growth Factor Receptor 2 (HER2) -Specific Chimeric Antigen Receptor-Modified T Cells for the Immunotherapy of HER2-Positive Sarcoma. *Journal of clinical oncology : official journal of the American Society of Clinical Oncology* **33:** 1688–1696

Beatty GL, Haas AR, Maus MV, Torigian DA, Soulen MC, Plesa G, Chew A, Zhao Y, Levine BL, Albelda SM, Kalos M & June CH (2014) Mesothelin-specific chimeric antigen receptor mRNA-engineered T cells induce anti-tumor activity in solid malignancies. *Cancer immunology research* **2:** 112–120

Brown CE, Badie B, Barish ME, Weng L, Ostberg JR, Chang W-C, Naranjo A, Starr R, Wagner J, Wright C, Zhai Y, Bading JR, Ressler JA, Portnow J, D’Apuzzo M, Forman SJ & Jensen MC (2015) Bioactivity and Safety of IL13Ralpha2-Redirected Chimeric Antigen Receptor CD8+ T Cells in Patients with Recurrent Glioblastoma. *Clinical cancer research : an official journal of the American Association for Cancer Research* **21:** 4062–4072

Feng K, Guo Y, Dai H, Wang Y, Li X, Jia H & Han W (2016) Chimeric antigen receptor-modified T cells for the immunotherapy of patients with EGFR-expressing advanced relapsed/refractory non-small cell lung cancer. *Science China. Life sciences* **59:** 468–479

Junghans RP, Ma Q, Rathore R, Gomes EM, Bais AJ, Lo ASY, Abedi M, Davies RA, Cabral HJ, Al-Homsi AS & Cohen SI (2016) Phase I Trial of Anti-PSMA Designer CAR-T Cells in Prostate Cancer: Possible Role for Interacting Interleukin 2-T Cell Pharmacodynamics as a Determinant of Clinical Response. *The Prostate* **76:** 1257–1270

Kandalaft LE, Powell DJ, JR & Coukos G (2012) A phase I clinical trial of adoptive transfer of folate receptor-alpha redirected autologous T cells for recurrent ovarian cancer. *Journal of translational medicine* **10:** 157

Katz SC, Burga RA, McCormack E, Wang LJ, Mooring W, Point GR, Khare PD, Thorn M, Ma Q, Stainken BF, Assanah EO, Davies R, Espat NJ & Junghans RP (2015) Phase I Hepatic Immunotherapy for Metastases Study of Intra-Arterial Chimeric Antigen Receptor-Modified T-cell Therapy for CEA+ Liver Metastases. *Clinical cancer research : an official journal of the American Association for Cancer Research* **21:** 3149–3159

Kershaw MH, Westwood JA, Parker LL, Wang G, Eshhar Z, Mavroukakis SA, White DE, Wunderlich JR, Canevari S, Rogers-Freezer L, Chen CC, Yang JC, Rosenberg SA & Hwu P (2006) A phase I study on adoptive immunotherapy using gene-modified T cells for ovarian cancer. *Clinical cancer research : an official journal of the American Association for Cancer Research* **12:** 6106–6115

Koneru M, O’Cearbhaill R, Pendharkar S, Spriggs DR & Brentjens RJ (2015) A phase I clinical trial of adoptive T cell therapy using IL-12 secreting MUC-16(ecto) directed chimeric antigen receptors for recurrent ovarian cancer. *Journal of translational medicine* **13:** 102

Lamers CH, Sleijfer S, van Steenbergen S, van Elzakker P, van Krimpen B, Groot C, Vulto A, den Bakker M, Oosterwijk E, Debets R & Gratama JW (2013) Treatment of metastatic renal cell carcinoma with CAIX CAR-engineered T cells: clinical evaluation and management of on-target toxicity. *Molecular therapy : the journal of the American Society of Gene Therapy* **21:** 904–912

Lamers CHJ, Langeveld SCL, Groot-van Ruijven CM, Debets R, Sleijfer S & Gratama JW (2007) Gene-modified T cells for adoptive immunotherapy of renal cell cancer maintain transgene-specific immune functions in vivo. *Cancer immunology, immunotherapy : CII* **56:** 1875–1883

Lamers CHJ, Sleijfer S, Vulto AG, Kruit WHJ, Kliffen M, Debets R, Gratama JW, Stoter G & Oosterwijk E (2006) Treatment of metastatic renal cell carcinoma with autologous T-lymphocytes genetically retargeted against carbonic anhydrase IX: first clinical experience. *Journal of clinical oncology : official journal of the American Society of Clinical Oncology* **24:** e20-2

Lamers CHJ, Willemsen R, van Elzakker P, van Steenbergen-Langeveld S, Broertjes M, Oosterwijk-Wakka J, Oosterwijk E, Sleijfer S, Debets R & Gratama JW (2011) Immune responses to transgene and retroviral vector in patients treated with ex vivo-engineered T cells. *Blood* **117:** 72–82

Louis CU, Savoldo B, Dotti G, Pule M, Yvon E, Myers GD, Rossig C, Russell HV, Diouf O, Liu E, Liu H, Wu M-F, Gee AP, Mei Z, Rooney CM, Heslop HE & Brenner MK (2011) Antitumor activity and long-term fate of chimeric antigen receptor-positive T cells in patients with neuroblastoma. *Blood* **118:** 6050–6056

Maus MV, Haas AR, Beatty GL, Albelda SM, Levine BL, Liu X, Zhao Y, Kalos M & June CH (2013) T cells expressing chimeric antigen receptors can cause anaphylaxis in humans. *Cancer immunology research* **1:** 26–31

Morgan RA, Yang JC, Kitano M, Dudley ME, Laurencot CM & Rosenberg SA (2010) Case report of a serious adverse event following the administration of T cells transduced with a chimeric antigen receptor recognizing ERBB2. *Molecular therapy : the journal of the American Society of Gene Therapy* **18:** 843–851

Park JR, Digiusto DL, Slovak M, Wright C, Naranjo A, Wagner J, Meechoovet HB, Bautista C, Chang W-C, Ostberg JR & Jensen MC (2007) Adoptive transfer of chimeric antigen receptor re-directed cytolytic T lymphocyte clones in patients with neuroblastoma. *Molecular therapy : the journal of the American Society of Gene Therapy* **15:** 825–833

Petrausch U, Schuberth PC, Hagedorn C, Soltermann A, Tomaszek S, Stahel R, Weder W & Renner C (2012) Re-directed T cells for the treatment of fibroblast activation protein (FAP)-positive malignant pleural mesothelioma (FAPME-1). *BMC Cancer* **12:** 615

Pule MA, Savoldo B, Myers GD, Rossig C, Russell HV, Dotti G, Huls MH, Liu E, Gee AP, Mei Z, Yvon E, Weiss HL, Liu H, Rooney CM, Heslop HE & Brenner MK (2008) Virus-specific T cells engineered to coexpress tumor-specific receptors: persistence and antitumor activity in individuals with neuroblastoma. *Nature medicine* **14:** 1264–1270

Saied A, Licata L, Burga RA, Thorn M, McCormack E, Stainken BF, Assanah EO, Khare PD, Davies R, Espat NJ, Junghans RP & Katz SC (2014) Neutrophil:lymphocyte ratios and serum cytokine changes after hepatic artery chimeric antigen receptor-modified T-cell infusions for liver metastases. *Cancer gene therapy* **21:** 457–462

van Schalkwyk MCI, Papa SE, Jeannon J-P, Guerrero Urbano T, Spicer JF & Maher J (2013) Design of a phase I clinical trial to evaluate intratumoral delivery of ErbB-targeted chimeric antigen receptor T-cells in locally advanced or recurrent head and neck cancer. *Human gene therapy. Clinical development* **24:** 134–142

You F, Jiang L, Zhang B, Lu Q, Zhou Q, Liao X, Wu H, Du K, Zhu Y, Meng H, Gong Z, Zong Y, Huang L, Lu M, Tang J, Li Y, Zhai X, Wang X, Ye S & Chen D et al (2016) Phase 1 clinical trial demonstrated that MUC1 positive metastatic seminal vesicle cancer can be effectively eradicated by modified Anti-MUC1 chimeric antigen receptor transduced T cells. *Science China. Life sciences* **59:** 386–397

1. [?] Information about CAR T cell clinical trials collected from ClinicalTrials.gov or literature. 78 CAR T cell clinical trials for solid tumors were registered by the end of 2016. Depicted are the status of each trial (ongoing, completed; terminated; suspended; withdrawn; unknown), the targeted antigen, the treated indication, the phase of the trial (phase I, phase I/II, Phase II), the number and age of enrolled patients, the generation of the CAR constructed used, whether a pre-treatment like lymphodepletion or chemotherapy, systemic IL-2 administration or a dose escalation regimen were applied, the sponsor of the trial, the country of the trial site/s, the identifier and follow-up time. [↑](#footnote-ref-2)
