# Supplementary material for: Clinical development of CAR T cells—challenges and opportunities in translating innovative treatment concepts
Source: EMBO Mol Med. 2017 Aug 1;9(9):1183–97. doi: 10.15252/emmm.201607485 (PMC5582407; doi:10.15252/emmm.201607485)
Supplement: Supplementary file 4 — Dataset EV3 [file EMMM-9-1183-s004.doc]

## Dataset EV3. Details from published CAR gene therapy clinical trials for solid tumors (13 total)[**[[1]](#footnote-2)**]

| **Antigen (Ab clone)**  **Indication** | **T cell origin (SCT)**  **pre-treatment**  **IL2 administration** | **CAR construct**  **Vector %CAR+ cells**  (median; range) | **T cell dose**  **Persistence** (detection) | ***n***  **age**  (median; range) | **Phase**  **Outcome *n***  (median; range)  in month | **Toxicities** | **Identifier**  **(Ref), Status** |
| --- | --- | --- | --- | --- | --- | --- | --- |
| CAIX (G250)  RCC | autologous - PT | scFv/CD3z  RV  53 (24-63) | 0.2-2.1x10^9^  (split dose over 2x5 days)  up to 4 weeks (PCR) | 12  62,5 (46-74) | Phase I  NE: 12 | - Four patients developed transient liver enzyme disturbances (grade 3/4). The toxicity was caused by antigen-specific encounter of the CAR T cells with CAIX expressing bile duct epithelial cells (on-target toxicity) | DDHK97-29/P00.0040C  (Lamers et al, 2013; Lamers et al, 2011; Lamers et al, 2007; Lamers et al, 2006), terminated |
| CEA (hMN14)  Liver metastases | autologous  +/- IL2 | scFv/CD28/CD3z  RV  53 (10-64) | 10^8^-10^10^  (single hepatic artery infusion) | 7  53,5 (51-66) | Phase I  SD: 1 (23) PD: 5 NE: 1 | - One patient experienced grade 3 fever and tachycardia, associated to high dose IL-2 administration. After a 50% dose reduction in systemic IL-2 infusion the fever and tachycardia resolved. - All patients experienced grade 1/2 transient elevations of alkaline phosphatase, total bilirubin and aspartate aminotransferase levels, but only patient developed grade 3 elevations | NCT01373047  (Katz et al, 2015; Saied et al, 2014), completed |
| EGFR  EGFR+ solid tumors | autologous +/- PT | scFv/4-1BB/CD3z  LV  29 (21-32) | 4x10^5^-2.54x10^7^/kg  up to 37 weeks (PCR) | 11  58 (40-66) | Phase I  PR: 2 (3; 2-3,5) SD: 5 (5,5; 2-8+) PD: 4 | - The most common grade 1/2 adverse event were mild skin toxicity (2), nausea (1), vomiting (1), dyspnea (4) and hypotension (1) - One patient suffered from a transient grade 3/4 increase in serum lipase | NCT01869166  (Feng et al, 2016), ongoing |
| ErbB2/Her2 (4D5)  Metastatic Cancer | autologous  + IL2 | scFv/CD28/4-1BB/  CD3z  RV  79 | 1x10^10^ total | 1  39 | Phase I/II  death: 1 | - **One patient died** within 5 days after CAR T cell infusion. Rapidly after cell infusion, the patient developed respiratory distress and showed dramatic pulmonary infiltrate on chest X-ray. The patient developed severe hypotension and experienced bradycardia as well as gastrointestinal bleeding which resulted in a cardiac arrest from which the patient could not be resuscitated. The death was most likely caused by on-target off-tumor toxicity associated with a rapid cytokine storm. Upon first-pass clearance in the lung, the CAR T cells recognized ErbB2 expressed by normal lung cells and released inflammatory cytokines that caused pulmonary toxicity and edema followed by a cascading cytokine storm resulting in multiorgan failure. | NCT00924287  (Morgan et al, 2010), terminated |
| ErbB2/Her2 (FRP5)  Sarcoma | autologous - PT | scFv/CD28/CD3z  RV  65 (36-88) | 1x10^4^-1x10^8^/m^2^  (up to 9 infusions)  up to 18 month (PCR) | 19  16 (7-29) | Phase I/II  SD: 4 (0,5; 0,5-14) PD: 13 NE: 2 | - One patient on the highest dose level developed fever within 12 hours after T cell infusion | NCT00902044  (Ahmed et al, 2015), ongoing |
| FR-a (MOv18)  Ovarian cancer | autologous  +/- IL2 | scFv/FcRγ  RV  low level | 0.3-5x10^10^ T cells  up to >1 year (PCR) | 14  (33-60) | Phase I  NE: 14 | - Some patients experienced grade 3/4 toxicities including hypotension and dyspnea as well as less frequently fatigue, leukopenia, rigors, sinus tachycardia and diarrhea, which was most likely due to IL-2 administration, because patients without IL-2 administration experienced only relatively mild side effects | (Kershaw et al, 2006), terminated |
| GD2 (14g2a)  Neuroblastoma | autologous - PT | RV | 1.2x10^7^-1x10^8^/m^2^ (single infusion)  up to 192 weeks for ATC and 96 weeks for CTLs (PCR) | 19[[[2]](#footnote-3)]  7 (3-29) | Phase I  CR: 11 PR: 1 SD: 1 PD: 6 | - Two patients suffered from mild to moderate local pain at the site of tumor necrosis, attributed to CAR T cell related killing of tumor cells - One patient experienced unexplained local pain | NCT00085930  (Louis et al, 2011; Pule et al, 2008), ongoing |
| IL-13Ra2  brain and CNS tumors | autologous - PT | ligand/CD3z  EP | 1.6x10^8^ for 1st cycle + 3x10^8^ for 2nd-4th cycle (split dose in each cycle; 3x infusions 2 days apart)  up to 14 weeks (IHC) | 3  57 (36-57) | Phase I  PR: 2 (12; 10-14) PD: 1 | - Two patients suffered from grade 3 headache, which was associated with CAR T cell infusion at a high dose (10^8^ cells) - One patient experienced grade 3 neurologic adverse events including shuffling gait and tongue deviation | NCT00730613  (Brown et al, 2015), completed |
| L1-CAM  (CE7R)  Neuroblastoma | autologous - PT | scFv/CD3z  EP | 1x10^8^-1.1x10^9^/m^2^ (split dose; 2-3x infusions 14 days apart)  up to 42 days (PCR) | 6[[[3]](#footnote-4)]  9,5 (7-16) | Phase I  CR: 1 (1,5) SD: 1 (1) PD: 4 | - Some patients suffered from grade 3 lymphopenia, neutropenia, low hemoglobin and bacteremia, which was associated with 10^8^/m^2^ CAR T cells infusion - One patient experienced grade 3 pneumonitis, which was associated with 10^9^/m^2^ CAR T cells infusion | NCT00006480  (Park et al, 2007), completed |
| Mesothelin  MPM | autologous - PT | scFv/4-1BB/CD3z  EP  99 (98-99,5) | 3x i.v. infusions with 0.1-1x10^9^ cells or 8x i.v. infusions with 3x10^8^/m^2^ and 2x i.t. infusions with 2x10^8^  transient CAR expression | 2  78 (81-75) | Phase I  PR: 1 (6) SD: 1 | - Patient 17510-105 suffered from an anaphylactic reaction shortly after the third CAR T cell infusion leading to grade 4 cardiac arrest, respiratory failure, disseminated intravenous coagulation and CRS associated with systemic mast cell degranulation related to an antibody response directed against the murine-derived scFv CAR construct - Patient 21211-101 developed grade 4 jejunal obstruction, grade 3 abdominal pain and grade 2 lymphcytosis. Also for this patient a IgG human anti-chimeric antibody response became detectable 64 days after the last cell infusion | NCT01355965  (Beatty et al, 2014; Maus et al, 2013), ongoing |
| MUC1  (SM3)  HCC, NSCLC, TNBC, PC | autologous - PT | scFv/CD28/4-1BB/  CD3z +/- IL-12 co-expression  LV  19,5 (18-21) | 5x10^5^ CAR T cells per lesion | 1 | Phase I  PR: 1 | - The patient suffered from mild headache, muscle pain, nasal congestion and abdominal bloating discomfort. - In addition, a transient CRS was experienced | NCT02587689  (You et al, 2016), ongoing |
| PSMA  Prostate Cancer | autologous + PT + IL2 | scFv/CD3z  RV  40 (29-61) | 1x10^9^ – 1x10^10^ total T cells  up to 4 week (FACS) | 5  61 (51-75) | Phase I  PR: 3 NE: 2 | - All patients experienced grade 3/4 hematologic toxicities including neutropenia, neutropenic fever and thrombocytopenia. In one case also anemia, hypocalcemia, hypophosphatemia and appendicitis were reported. - Some patients showed grade 1/2 skin rash, fatigue, intermittent low-grade fevers and muscle pain attributable to systemic IL-2 infusions | BB-IND 12084  (Junghans et al, 2016), terminated |
| VEGFR-2  various | autologous + PT + IL2 | RV |  | 23 | Phase I/II  PR: 1 SD: 1 PD: 21 | - **One patient** died due to an infection (most likely not related to CAR T cell infusion) - Some patients showed grade 3/4 toxicity include nausea (1), vomiting (1), hypoxia (2) and elevated levels of aspartate transaminase (3), alanine transaminase (3) and bilirubin (3) | NCT01218867  (Rosenberg, 2016), completed |

**Ab**, antibody; ***n****,* number of treated patients; **PT**, pretreatment like lymphodepletion or chemotherapy; **IL2**, systemic IL-2 administration; **+**, yes; **-**, no; **+/-**, variable; **RV**, retroviral vector; **LV**, Lentiviral vector; **EP**, electroporation; **CR**, complete response; **PR**, partial response; **SD**, stable disease; **PD**, progressive disease; **NE**, no response**; CAIX**, carbonic anhydrase IX; **CEA**, carcinoembryonic antigen; **c-MET**, hepatocyte growth factor receptor; **EGFR**, epidermal growth factor receptor; **ErbB2/Her2**, human epidermal growth factor receptor 2; **FR-a**, Folate receptor alpha; **IL-13Ra2**, Interleukin-13 receptor subunit alpha-2; **MUC1**, Mucin 1; **PSMA**, prostate specific membrane antigen; **VEGFR-2**, Vascular endothelial growth factor receptor 2; **MPM**, malignant pleural mesothelioma; **RCC**, renal cell carcinoma

References

Ahmed N, Brawley VS, Hegde M, Robertson C, Ghazi A, Gerken C, Liu E, Dakhova O, Ashoori A, Corder A, Gray T, Wu M-F, Liu H, Hicks J, Rainusso N, Dotti G, Mei Z, Grilley B, Gee A & Rooney CM et al (2015) Human Epidermal Growth Factor Receptor 2 (HER2) -Specific Chimeric Antigen Receptor-Modified T Cells for the Immunotherapy of HER2-Positive Sarcoma. *Journal of clinical oncology : official journal of the American Society of Clinical Oncology* **33:** 1688–1696

Beatty GL, Haas AR, Maus MV, Torigian DA, Soulen MC, Plesa G, Chew A, Zhao Y, Levine BL, Albelda SM, Kalos M & June CH (2014) Mesothelin-specific chimeric antigen receptor mRNA-engineered T cells induce anti-tumor activity in solid malignancies. *Cancer immunology research* **2:** 112–120

Brown CE, Badie B, Barish ME, Weng L, Ostberg JR, Chang W-C, Naranjo A, Starr R, Wagner J, Wright C, Zhai Y, Bading JR, Ressler JA, Portnow J, D’Apuzzo M, Forman SJ & Jensen MC (2015) Bioactivity and Safety of IL13Ralpha2-Redirected Chimeric Antigen Receptor CD8+ T Cells in Patients with Recurrent Glioblastoma. *Clinical cancer research : an official journal of the American Association for Cancer Research* **21:** 4062–4072

Feng K, Guo Y, Dai H, Wang Y, Li X, Jia H & Han W (2016) Chimeric antigen receptor-modified T cells for the immunotherapy of patients with EGFR-expressing advanced relapsed/refractory non-small cell lung cancer. *Science China. Life sciences* **59:** 468–479

Junghans RP, Ma Q, Rathore R, Gomes EM, Bais AJ, Lo ASY, Abedi M, Davies RA, Cabral HJ, Al-Homsi AS & Cohen SI (2016) Phase I Trial of Anti-PSMA Designer CAR-T Cells in Prostate Cancer: Possible Role for Interacting Interleukin 2-T Cell Pharmacodynamics as a Determinant of Clinical Response. *The Prostate* **76:** 1257–1270

Katz SC, Burga RA, McCormack E, Wang LJ, Mooring W, Point GR, Khare PD, Thorn M, Ma Q, Stainken BF, Assanah EO, Davies R, Espat NJ & Junghans RP (2015) Phase I Hepatic Immunotherapy for Metastases Study of Intra-Arterial Chimeric Antigen Receptor-Modified T-cell Therapy for CEA+ Liver Metastases. *Clinical cancer research : an official journal of the American Association for Cancer Research* **21:** 3149–3159

Kershaw MH, Westwood JA, Parker LL, Wang G, Eshhar Z, Mavroukakis SA, White DE, Wunderlich JR, Canevari S, Rogers-Freezer L, Chen CC, Yang JC, Rosenberg SA & Hwu P (2006) A phase I study on adoptive immunotherapy using gene-modified T cells for ovarian cancer. *Clinical cancer research : an official journal of the American Association for Cancer Research* **12:** 6106–6115

Lamers CH, Sleijfer S, van Steenbergen S, van Elzakker P, van Krimpen B, Groot C, Vulto A, den Bakker M, Oosterwijk E, Debets R & Gratama JW (2013) Treatment of metastatic renal cell carcinoma with CAIX CAR-engineered T cells: clinical evaluation and management of on-target toxicity. *Molecular therapy : the journal of the American Society of Gene Therapy* **21:** 904–912

Lamers CHJ, Langeveld SCL, Groot-van Ruijven CM, Debets R, Sleijfer S & Gratama JW (2007) Gene-modified T cells for adoptive immunotherapy of renal cell cancer maintain transgene-specific immune functions in vivo. *Cancer immunology, immunotherapy : CII* **56:** 1875–1883

Lamers CHJ, Sleijfer S, Vulto AG, Kruit WHJ, Kliffen M, Debets R, Gratama JW, Stoter G & Oosterwijk E (2006) Treatment of metastatic renal cell carcinoma with autologous T-lymphocytes genetically retargeted against carbonic anhydrase IX: first clinical experience. *Journal of clinical oncology : official journal of the American Society of Clinical Oncology* **24:** e20-2

Lamers CHJ, Willemsen R, van Elzakker P, van Steenbergen-Langeveld S, Broertjes M, Oosterwijk-Wakka J, Oosterwijk E, Sleijfer S, Debets R & Gratama JW (2011) Immune responses to transgene and retroviral vector in patients treated with ex vivo-engineered T cells. *Blood* **117:** 72–82

Louis CU, Savoldo B, Dotti G, Pule M, Yvon E, Myers GD, Rossig C, Russell HV, Diouf O, Liu E, Liu H, Wu M-F, Gee AP, Mei Z, Rooney CM, Heslop HE & Brenner MK (2011) Antitumor activity and long-term fate of chimeric antigen receptor-positive T cells in patients with neuroblastoma. *Blood* **118:** 6050–6056

Maus MV, Haas AR, Beatty GL, Albelda SM, Levine BL, Liu X, Zhao Y, Kalos M & June CH (2013) T cells expressing chimeric antigen receptors can cause anaphylaxis in humans. *Cancer immunology research* **1:** 26–31

Morgan RA, Yang JC, Kitano M, Dudley ME, Laurencot CM & Rosenberg SA (2010) Case report of a serious adverse event following the administration of T cells transduced with a chimeric antigen receptor recognizing ERBB2. *Molecular therapy : the journal of the American Society of Gene Therapy* **18:** 843–851

Park JR, Digiusto DL, Slovak M, Wright C, Naranjo A, Wagner J, Meechoovet HB, Bautista C, Chang W-C, Ostberg JR & Jensen MC (2007) Adoptive transfer of chimeric antigen receptor re-directed cytolytic T lymphocyte clones in patients with neuroblastoma. *Molecular therapy : the journal of the American Society of Gene Therapy* **15:** 825–833

Pule MA, Savoldo B, Myers GD, Rossig C, Russell HV, Dotti G, Huls MH, Liu E, Gee AP, Mei Z, Yvon E, Weiss HL, Liu H, Rooney CM, Heslop HE & Brenner MK (2008) Virus-specific T cells engineered to coexpress tumor-specific receptors: persistence and antitumor activity in individuals with neuroblastoma. *Nature medicine* **14:** 1264–1270

Rosenberg S (2016) *CAR T Cell Receptor Immunotherapy Targeting VEGFR2 for Patients With Metastatic Cancer.* <https://clinicaltrials.gov/ct2/show/results/NCT01218867>

Saied A, Licata L, Burga RA, Thorn M, McCormack E, Stainken BF, Assanah EO, Khare PD, Davies R, Espat NJ, Junghans RP & Katz SC (2014) Neutrophil:lymphocyte ratios and serum cytokine changes after hepatic artery chimeric antigen receptor-modified T-cell infusions for liver metastases. *Cancer gene therapy* **21:** 457–462

You F, Jiang L, Zhang B, Lu Q, Zhou Q, Liao X, Wu H, Du K, Zhu Y, Meng H, Gong Z, Zong Y, Huang L, Lu M, Tang J, Li Y, Zhai X, Wang X, Ye S & Chen D et al (2016) Phase 1 clinical trial demonstrated that MUC1 positive metastatic seminal vesicle cancer can be effectively eradicated by modified Anti-MUC1 chimeric antigen receptor transduced T cells. *Science China. Life sciences* **59:** 386–397

1. [?] Data were collected from 13 CAR T cell clinical trials for solid tumors published by the end of 2016. In the first column the targeted antigen, the antibody clone of the scFv and the treated indication are described. The second column provides information about the T cell origin, and whether stem cell transplantation, a pre-treatment like lymphodepletion, or chemotherapy, or systemic IL-2 administration were applied. In the third column the domain organisation of the CAR construct, the used vector to generate CAR T cells as well as the percentage of generated CAR T cells are mentioned. In the next column information about the applied T cell dose and number of infusions is provided as well as information about CAR T cells persistence (longest observed time frame in single patients) and the applied detection method. In addition, information about the number, age and clinical outcome of treated patient as well as the phase of the trial and observed toxicities are provided. In the last column, the trial identifier, publication reference and the status of the trial (ongoing, completed; terminated; suspended; unknown) are indicated. Information about disease status is provided in further footnotes. [↑](#footnote-ref-2)
2. [?] At time of CAR T cell infusion, 8 patients had no evidence of active disease, 4 patients had limited disease and 7 patients had active, bulky disease. The 8 patients with no evidence of active disease showed no evidence of disease as best clinical outcome and 3 patients with limited disease ended up with complete response as best clinical outcome. [↑](#footnote-ref-3)
3. [?] One patient had only limited disease burden upon study enrollment. This patient achieved a partial response 56 days after CAR T cell administration, but showed progressive disease after additional therapy. [↑](#footnote-ref-4)
