# Supplementary material for: Clinical development of CAR T cells—challenges and opportunities in translating innovative treatment concepts
Source: EMBO Mol Med. 2017 Aug 1;9(9):1183–97. doi: 10.15252/emmm.201607485 (PMC5582407; doi:10.15252/emmm.201607485)
Supplement: Supplementary file 5 — Dataset EV4 [file EMMM-9-1183-s005.doc]

## Dataset EV4. Details from published CAR gene therapy clinical trials for hematological malignancies (29 total)[**[[1]](#footnote-2)**]

| **Antigen (Ab clone)**  **Indication** | **T cell origin (SCT)**  **pre-treatment**  **IL2 administration** | **CAR construct**  **Vector %CAR+ cells**  (median; range) | **T cell dose** (dose/infusion)  **Persistence** (detection) | ***n***  **age**  (median; range) | **Phase**  **Outcome *n***  (median; range)  in month | **Toxicities** | **Identifier**  **(Ref), Status** |
| --- | --- | --- | --- | --- | --- | --- | --- |
| BCMA (11D5-3)  MM | autologous + PT | scFv/CD28/CD3z  RV  67 (45-93) | 0.3-9x10^6^/kg  (single dose)  up to 3 month (PCR) | 12 | Phase I  CR: 1 (17) PR: 3 (8; 2-26+) SD: 8 (6; 2-12) | - All patients experienced grade 3/4 cytopenias which could be attributed to the conditioning regimen or when prolong, as observed in two patients, rather to the CAR T cell treatment (highest cell dose) - Two patients exhibited grade 2/3 signs and symptoms of CRS including mild fever and tachycardia - One patient had significant fever, tachycardia and hypotension among other grade 2/3 toxicities - Two patients had grade 3/4 toxicity consistent with CRS among others | NCT02215967  (Ali et al, 2016), ongoing |
| CD138  MM | autologous - PT | scFv/4-1BB/CD3z  LV  34 (12-41) | 0.44-3.78x10^7^/kg  up to >5 month (PCR) | 5  57 (48-68) | Phase I  SD: 4 (4,5; 3-7)  PD: 1 | - all patients suffered from transient grade 3 chills and fever, but recovered overnight - 3 patients developed grade 2 TLS with nausea and vomiting, which was associated with elevated CAR T cell levels in the peripheral blood | NCT01886976  (Guo et al, 2016), ongoing |
| CD19  NHL | autologous (+) + PT | scFv/CD28/CD3z  EP(SB)  88,5 (77-96) | 5x10^8^-5x10^9^/m^2^  (single and split-infusion over 2-3 days)  average of 201 days (PCR) | 7[[[2]](#footnote-3)]  55 (36-61) | Phase I  CR: 6 (26; 6-33) PD: 1 |  | NCT00968760  (Kebriaei et al, 2016), ongoing |
| CD19  Leukemia, Lymphoma | allogenic (+) - PT | scFv/CD28/CD3z  EP(SB)  83 (59-97) | 1x10^6^-5x10^8^/m^2^  (single and split-infusion over 2-3 days)  average of 51 days (PCR), in one patient detectable up to 1 year | 19[[[3]](#footnote-4)]  36 (21-56) | Phase I  CR: 10 (8; 3-18) PD: 9 | - Three patients developed GVHD:  1 grade 1 acute skin GVHD,  1 chronic skin GVHD and  1 liver GVHD | NCT01497184  (Kebriaei et al, 2016), ongoing |
| CD19 (FMC63)  DLBCL | autologous + PT | scFv/CD3z  EP | 4-5 infusions with 1x10^8^-2x10^9^/m^2^ each  1 day (PCR) | 2 | Phase I  PD: 2 |  | IRB 01160  (Jensen et al, 2010), completed |
| CD19 (FMC63)  DLBCL, PBMCL, DLBCL transf. FL, MCL | autologous + PT +/- IL2 | scFv/CD28/CD3z  RV  70 (54-84) | 3x10^6^-3x10^7^/kg  (single infusion)  up to 14 weeks (PCR) | 21  55 (30-68) | Phase I  CR: 8 (13; 6-23+) PR: 8 (7; 1-23+) SD: 2 (3,5; 1-6) NE: 3 | - **Two patients died** (most likely not related to CAR T cell infusion):   Patient 4b suddenly died 16 days after cell infusion with no signs of CRS, most likely not related to CAR T cell administration  Patient 2a died 18 days after cell infusion of influenza pneumonia, nonbacterial thrombotic endocarditis, and cerebral infarction most likely not related to CAR T cell administration   - 19 patients developed acute grade 3/4 toxicities including fever, hypotension, delirium, and other neurologic toxicities. These toxicities occurred mostly during first 2 weeks after infusion and resolved within 3 weeks after cell infusion - Three patients showed B cell aplasia for at least 4 month after CAR T cell infusion. Consistent with the in vivo presence of functional CD19 CAR–T cells. Other patients were not evaluated for B cell depletion due to prior rituximab treatment. | NCT00924326  (Kochenderfer et al, 2015; Kochenderfer et al, 2012; Kochenderfer et al, 2010), ongoing |
| CD19 (FMC63)  NHL, ALL, CLL | allogenic VSTs (+) - PT | scFv/CD28/CD3z  RV  (20-48) | 1.5x10^7^-1,2x10^8^/m^2^ total T cells (single infusion; 1-3x doses)  up to 12 weeks (PCR) | 8[[[4]](#footnote-5)]  44,5 (9-59) | Phase I  CR: 3 (3; 2-8+) PR: 1 (2) SD: 1 (15+) PD: 3 |  | NCT00840853  (Cruz et al, 2013), ongoing |
| CD19 (FMC63)  ALL, CLL, DLBCL, FL, MCL, PLL | autologous + PT | scFv/4-1BB/CD3z  LV  20 (5-39) | 2x10^7^-1x10^8^/m^2^ (single infusion)  up to 4 years (PCR) | 14  66 (51-78) | Phase *NA*  CR: 5 (28; 10-53) PR: 3 (6; 5-13) NE: 6 (3,5; 1-10) | - Six patients developed persistence B cell aplasia for up to 1 year, consistent with the in vivo presence of functional CD19-CAR T cells and a positive clinical outcome - Nine patients developed mild to severe CRS 1 to 14 days after cell infusion, which was associated with a higher peak expansion of CAR T cells - Five patients showed grade 1-4 neurological events - Two cases of TLS were noted | NCT01029366  (Maude et al, 2014; Kalos et al, 2011; Porter et al, 2011; Porter et al, 2015), completed |
| CD19 (FMC63)  NHL, HL | allogenic (+) - PT | scFv/CD28/CD3z  RV  58 (34-78) | 0.4-7.8x10^6^/kg  (single infusion)  up to 1 month (PCR) | 10[[[5]](#footnote-6)]  49,5 (44-66) | Phase I  CR: 1 (9+) PR: 1 (3+) SD: 6 (3; 1-11+) PD: 2 | - Three patients developed transient grade 3/4 B cell aplasia, consistent with the in vivo presence of functional CD19 CAR–T cells - Patient 3 showed grade 3/4 TLS, fatigue, cardiac ventricular dysfunction, fever, tachycardia, troponin increase, anemia and neutropenia. The etiology of patient 3’s toxicities is unclear because he had pulmonary infiltrates and mild dyspnea before his CAR T-cell infusion - Two patients experienced grade 3/4 CRS associated toxicities including hypotensions - all toxicities experienced (except patient 1) resolved completely in <2 weeks after CAR T-cell infusion. - no patient developed sings of GVHD | NCT01087294  (Kochenderfer et al, 2013), suspended |
| CD19 (FMC63)  ALL, FL, CLL, MCL, DLBCL, PLL | autologous + PT | scFv/4-1BB/CD3z  LV  21,5 (5,5-45) | 0.76-20.6x10^6^/kg  (split dose over 1-3 days)  up to 6 month (PCR) | 30[[[6]](#footnote-7)]  11 (5-22) | Phase I/II  CR: 27 NE: 3 | - All patients had CRS, 8 patients developed severe CRS which was associated with a higher disease burden before infusion - 13 patients showed self-limiting signs of neurologic disorders lasting 2-3 days which was associated with CRs - 27 patients developed persistence B cell aplasia for up to 1 year, consistent with the in vivo presence of functional CD19-CAR T cells and a positive clinical outcome | NCT01626495  (Maude et al, 2014; Fitzgerald et al, 2016; Grupp et al, 2013), ongoing |
| CD19 (FMC63)  DLBCL, DMBCL, DSBCL, FL, MCL | autologous (+) + PT | scFv/CD3z  LV  84 (45-92) | 2.5 x10^7^-1x10^8^  (single infusion)  mean of 18.25 days (PCR) | 8[[[7]](#footnote-8)]  61,5 (50-75) | Phase I  CR: 5 (24; 6-37+) PR: 2 (18; 10-26+) PD: 1 |  | NCT01318317  (Wang et al, 2016b), ongoing |
| CD19 (FMC63)  ALL, DLBCL, NHL | autologous (+/-) + PT | scFv/CD28/CD3z  RV  66 (55-77) | 1-3x10^6^/kg  (single infusion)  up to 6 weeks (PCR) | 21[[[8]](#footnote-9)]  13 (1-30) | Phase I  CR: 14 SD: 3 PD: 4 | - 16 patients experienced mild to severe CRS; 6 patients developed grade 3/4 CRS. Notably, mild CRS was self-limiting for 4-8 days. - Six patients showed signs of neurotoxicity with CAR T cells detection in the CSF - Most patients had grade 3/4 cytopenias which were attributed to lymphodepleting chemotherapy - Two patients of four had dose-limiting toxicity (grade 3/4 CRS) receiving 3x10^6^ cells/kg - Nine patients developed grade 3 fever, hypokalemia or neutropenia | NCT01593696  (Lee et al, 2015), ongoing |
| CD19 (FMC63)  CLL, SLL | autologous | scFv/4-1BB/CD3z  LV |  | 3  61 (57-68) | Phase *NA*  CR: 1 PR: 2 |  | NCT01747486  (Fraietta et al, 2016), ongoing |
| CD19 (FMC63)  ALL, CLL, MCL, FL, PLL | autologous, allogeneic (+/-) +/- PT - IL2 | scFv/4-1BB/CD3z | 3.6x10^6^-1.27x10^7^/kg  (split dose over 3-5 days; up to two infusions)  up to 3 month (PCR) | 9  35 (15-65) | Phase I  CR: 5 (4,5; 2-8,5) PD: 2 NE: 1  death: 1 | - **One patient died** (most likely related to CAR T cell infusion):   Patient 4 succumbed from acute TLS 12 h after the second CAR T cell infusion due to extensive and bulky adenopahty   - Nearly all patients developed grade 1-3 chills and fever 1-2 h after cell infusion, which subsided overnight - Three patients developed grade 1/4 CRS. The severity of CRS was linked with the degree of tumor burden. - Two patients treated after allogenic HSCT suffered from grade 2/3 GVHD 3-4 weeks after cell infusion; One patient (patient 8) died shortly after receiving anti-GVHD therapy   Notably, none of the patients treated with autologous HSCT prior to CAR T cell infusion developed GVHD | NCT01864889  (Dai et al, 2015), ongoing |
| CD19 (FMC63)  ALL | autologous (+/-) + PT | scFv/4-1BB/CD3z  LV  CD4: 79,7 (50-96) CD8: 84,2 (13-96) | 1x10^5^-1.16x10^7^/kg CD4+ CAR T cells and 3x10^4^-1x10^7^ CD8+ CAR T cells  up to >8 month (PCR) | 29[[[9]](#footnote-10)]  40 (20-73) | Phase I/II  CR: 26 PD: 1 death: 2 | - **Two patients died** (most likely related to CAR T cell infusion):   Patient 6 developed severe CRS and multiorgan failure and died 3 days after CAR T cell infusion. Notable, he received a high CAR T cell dose without risk-adapted dosing for high tumor burden and was unresponsive to tocilizumab, etanercept and corticosteroids.  Patient 17 developed transient CRS and achieved a complete response, but finally died 122 days after CAR T cell infusion with irreversible neurologic toxicity   - 28 patients suffered from mild to severe CRS, 7 patients required intensive care. The seven patients exhibiting severe CRS had a high tumor burden and were administered a high CAR T cell does (>2x10^6^ CAR T cells/kg). - 15 patients exhibited grade 3/4 neurotoxicites, which completely resolved over days to weeks (except one patient). Notably, severe neurotoxicities correlated with severe CRS - all patients showed B cell aplasia, consistent with in vivo presence of functional CD19-CAR T cells - Five of 11 patients were treated with cyclophosphamide alone and developed an immune response against the CAR T cells. Of note, the scFv fragment of the CD19-CAR is derived from a murine origin - No GVHD was observed in the 11 patients receiving prior allogenic-HSCT | NCT01865617  (Turtle et al, 2016), ongoing |
| CD19 (FMC63)  NHL | autologous (+) + PT | scFv/CD28/CD3z  LV  61 (24-77) | 5 x10^7^-2x10^8^  (single infusion)  mean of 20.5 days (PCR) | 8[[[10]](#footnote-11)]  61,5 (23-71) | Phase I  CR: 8 (12; 6-14+) |  | NCT01815749  (Wang et al, 2016b), ongoing |
| CD19 (FMC63)  MM | autologous (+) + PT | scFv/4-1BB/CD3z  LV | 1-5x10^7^  up to 47 days (PCR) | 10  60,5 (48-68) | Phase I  CR: 1 PR: 3 PD: 4 NE: 2 | - Grade 3/4 neutropenia, thrombocytopenia, mucositis, which were transplantation related and resolved by day 100 after transplantation - One patient with grade 1 CRS - One patient with grade 3 enterocolitis due to GVHD | NCT02135406  (Garfall et al, 2015), ongoing |
| CD19 (FMC63)  NHL | autologous - PT | scFv/CD28/CD3z or scFV/CD3z  RV | 0.2-2x10^8^/m^2^  up to >6 weeks | 6  54,5 (46-59) | Phase *NA*  SD: 2 (6,5; 3-10) PD: 4 |  | (Savoldo et al, 2011), unknown |
| CD19 (SJ25C13)  CLL | autologous +/- PT - IL2 | scFv/CD28/CD3z  RV  (23-70) | 1.2-3x10^7^/kg (single dose) 0.4-1x10^7^/kg (split dose over 2 days)  up to 8 weeks (ICH) | 8  60 (44-66) | Phase I/II  SD: 2 (6; 4-8+) PD: 5 NE: 1 | - **One patient died** (most likely not related to CAR T cell infusion): - Patient CLL-4 experienced persistent fever, developed a sepsis-like syndrome with hypotension and renal failure and died within 48 hours of CAR T cell infusion. However, an infectious cause preceded CAR T cell infusion as evidenced by marked serum cytokine abnormalities - All patients experienced grade 1/2 rigors, chills and transient fever - Two patients developed grade 3 febrile neutropenia | NCT00466531  (Brentjens et al, 2011; Brentjens et al, 2010), ongoing |
| CD19 (SJ25C13)  ALL | autologous (+/-) + PT | scFv/CD28/CD3z  RV  16,8 (5-61) | 3x10^6^/kg  (split dose over 2 days)  up to 3 month (PCR) | 16[[[11]](#footnote-12)]  50 | Phase I  CR: 14 PD: 2 | - Six patients developed grade 3/4 CRS with fever and hypotension, which was associated with a higher disease burden before infusion - Four patients showed neurological complications including delirium and seizure-like activity, associated with CRS. CAR T cells were detected in the CSF for three patients. | NCT01044069  (Brentjens et al, 2011; Brentjens et al, 2013; Davila et al, 2014), ongoing |
| CD20 (HB-9645)  ALL, CLL, PPL, DLBCL, FL, MCL | autologous  - IL2 | scFv/4-1BB/CD3z  LV  33 (15-50) | split dose over 3-5 days  up to >5 month (PCR) | 7[[[12]](#footnote-13)]  65 (37-85) | Phase *NA*  CR: 1 (14+) PR: 4 (3,5; 3-6) PD: 1 NE: 1 | - **One patient** (UPN2) **died** due to massive alimentary tract hemorrhage 3 weeks after infusion. The death was attributed to disease burden and not to the CAR T cell administration. - One patient developed grade 4 chills and fever accompanied by temporary and well-tolerated respiratory distress Five patients experienced transient and well-tolerated grade 1/2 chills and fever - Four patients showed damage of normal tissue in sites around the lesions and organ dysfunction due to on-target off-tumor toxicity due to low level expression of CD20 on normal tissue - Four patients developed grade 1-3 CRS and related toxicities including febrile syndrome, serous cavity effusion, capillary leak syndrome among others. Severe cytokine-release syndrome was associated with a higher disease burden before infusion - One patient showed a sudden tumor lysis syndrome 8 weeks after CAR T cell infusion | NCT01735604  (Wang et al, 2014), ongoing |
| CD20 (Leu16)  - Leukemia, Lymphoma | autologous +/- PT +/- IL2 | scFv/CD3z  EP | 5x10^8^-4.4x10^9^/m^2^ (split dose; 2-3x infusions 2-5 days apart)  up to 9 weeks (PCR) | 7[[[13]](#footnote-14)]  47 (43-77) | Phase I  CR: 2 (8; 3-13) PR: 1 (3) SD: 4 (5,5; 3-12) | - Two patients suffered from grade 2 skin reaction during IL-2 administration | NCT00012207  (Till et al, 2008), completed |
| CD20 (Leu-16)  - FL | autologous (+) | scFv/CD3z  EP | 3x infusions with 1x10^8^-1x10^9^/m^2^ each  up to 1 week (PCR) | 2 | Phase I  SD: 1 PD: 1 | - One patient developed cellular immune response against the infused T cell products. The immune reaction was directed against neomycin phosphotransferase but not the CAR construct. | IRB 98142  (Jensen et al, 2010), completed |
| CD20 (Leu-16)  CLL, FL, MCL, SLL, MZL | autologous + PT + IL2 | scFv/CD28/4-1BB/CD3z  EP  low level | 4.4x10^9^/m^2^ (split dose; 3x infusions 2-5 days apart)  up to 1 year (PCR) | 3  63,5 (28-80) | Phase I  CR: 2 (18; 12-24) PR: 1 (12) | - One patient developed transient infusional symptoms - One patient developed fever and orthostatic hypotension which was attributed to the second CAR T cell infusion - A heavily pretreated 80-year old patient developed grade 4 cytopenia as well as alopecia and fatigue - Some patients suffered from dyspnea, fatigue and skin reactions as consequence of low dose IL-2 treatment | NCT00621452  (Till et al, 2012), completed |
| CD30 (Ber-H2)  NHL, HL | autologous + PT | scFv/4-1BB/CD3z  LV  48 (36-68) | 1.1-2.1x10^7^/kg (split dose over 3 to 5 days)  up to >8 month (PCR) | 18  31 (13-77) | Phase I  PR: 6 (5; 2-9) SD: 7 (5; 2-12) PD: 5 | - One patient (13) suffered from grade 3/4 left ventricular systolic dysfunction associated with previous megadose of adriamycin - One patient (15) developed elevated alanine and aspartate aminotransferase levels - One patient (5) experienced instantaneous psychiatric abnormalities of mild anxiety and delirium along with fever syndrome as well as joint swelling and urticaria | NCT02259556  (Wang et al, 2016a), ongoing |
| CD33 (H33Mel)  AML | autologous - PT | scFv/4-1BB/CD3z  LV  38 | 1.12x10^9^ (split dose over 4 days)  at least 2 month (PCR) | 1  41 | Phase *NA*  PR: 1 (0,5) | - The patients suffered from severe chills and fevers as well as drastic fluctuations of his pre-existing pancytopenia - The patient experienced a spontaneous and intermittent febrile syndrome and showed signs of low grade CRS | NCT01864902  (Wang et al, 2015), ongoing |
| Ig k  (CRL-1758)  - CLL, NHL, MM | autologous +/- PT | scFv/CD28/CD3z  RV  91 (70-95) | 9.0x10^7^-1.9x10^9^/m^2^  (up to 6 infusions)  up to 6 month (PCR) | 17  59 (43-75) | Phase I  CR: 2 (17; 2-32+) PR: 1 (3) SD: 6 (2; 2-24) NE: 8 | - Most patients had baseline B cell lymphopenia and poyclonal hypogammaglobulinemia which was associated with prior treatment and/or biology of their underlying disease | NCT00881920  (Ramos et al, 2016), ongoing |
| Lewis Y  MM, AML, MDS | autologous + PT | scFv/CD28/CD3z  RV  25 (14-39) | 1-5x10^8^  up to 10 month (PCR) | 4  70 (64-71) | Phase I  PR: 2 (11; 1,5-22) SD: 2 (3; 1-5) | - One patient developed a transient skin rash 4 days and 22 after infusion. Histology and immunohistochemistry showed infiltration of the skin with AML blasts. | NCT01716364  (Ritchie et al, 2013), ongoing |

**Ab**, antibody; ***n****,* number of treated patients; **PT**, pre-treatment like lymphodepletion or chemotherapy; **SCT**, stem cell transplantation; **IL2**, systemic IL-2 administration; **+**, yes; **-**, no; **+/-**, variable; **allo**, allogenic; **auto**, autologous; **RV**, retroviral vector; **LV**, Lentiviral vector; **EP**, electroporation; **CR**, complete response; **PR**, partial response; **SD**, stable disease; **PD**, progressive disease; **NE**, no response; **BCMA**, B-cell maturation antigen; **Ig k**, Ig kappa light cahin; **ALL**, acute lymphoid leukemia; **AML**, acute myeloid leukemia; **CLL**, chronic lymphocytic leukemia; **DLBCL**, diffuse large B cell lymphoma; **DMBCL**, diffuse medium B cell lymphoma; **DSBCL**, diffuse small B cell lymphoma; **FL**, follicular lymphoma; **HL**, Hodgkin lymphoma; **MCL**, mantle cell lymphoma; **MDS**, myelodysplastic sndrome; **MM**, multiple myeloma; **MZL**, marginal zone lymphomas; **NHL**, non-Hodgkin lymphoma; **PLL**, B cell prolymphocytic leukemia; **SLL**, small lymphocytic lymphoma; **SCT**, Sinobioway Cell Therapy

References

Ali SA, Shi V, Maric I, Wang M, Stroncek DF, Rose JJ, Brudno JN, Stetler-Stevenson M, Feldman SA, Hansen BG, Fellowes VS, Hakim FT, Gress RE & Kochenderfer JN (2016) T cells expressing an anti-B-cell maturation antigen chimeric antigen receptor cause remissions of multiple myeloma. *Blood* **128:** 1688–1700

Brentjens R, Yeh R, Bernal Y, Riviere I & Sadelain M (2010) Treatment of chronic lymphocytic leukemia with genetically targeted autologous T cells: case report of an unforeseen adverse event in a phase I clinical trial. *Molecular therapy : the journal of the American Society of Gene Therapy* **18:** 666–668

Brentjens RJ, Davila ML, Riviere I, Park J, Wang X, Cowell LG, Bartido S, Stefanski J, Taylor C, Olszewska M, Borquez-Ojeda O, Qu J, Wasielewska T, He Q, Bernal Y, Rijo IV, Hedvat C, Kobos R, Curran K & Steinherz P et al (2013) CD19-targeted T cells rapidly induce molecular remissions in adults with chemotherapy-refractory acute lymphoblastic leukemia. *Science translational medicine* **5:** 177ra38

Brentjens RJ, Riviere I, Park JH, Davila ML, Wang X, Stefanski J, Taylor C, Yeh R, Bartido S, Borquez-Ojeda O, Olszewska M, Bernal Y, Pegram H, Przybylowski M, Hollyman D, Usachenko Y, Pirraglia D, Hosey J, Santos E & Halton E et al (2011) Safety and persistence of adoptively transferred autologous CD19-targeted T cells in patients with relapsed or chemotherapy refractory B-cell leukemias. *Blood* **118:** 4817–4828

Cruz CRY, Micklethwaite KP, Savoldo B, Ramos CA, Lam S, Ku S, Diouf O, Liu E, Barrett AJ, Ito S, Shpall EJ, Krance RA, Kamble RT, Carrum G, Hosing CM, Gee AP, Mei Z, Grilley BJ, Heslop HE & Rooney CM et al (2013) Infusion of donor-derived CD19-redirected virus-specific T cells for B-cell malignancies relapsed after allogeneic stem cell transplant: a phase 1 study. *Blood* **122:** 2965–2973

Dai H, Zhang W, Li X, Han Q, Guo Y, Zhang Y, Wang Y, Wang C, Shi F, Zhang Y, Chen M, Feng K, Wang Q, Zhu H, Fu X, Li S & Han W (2015) Tolerance and efficacy of autologous or donor-derived T cells expressing CD19 chimeric antigen receptors in adult B-ALL with extramedullary leukemia. *Oncoimmunology* **4:** e1027469

Davila ML, Riviere I, Wang X, Bartido S, Park J, Curran K, Chung SS, Stefanski J, Borquez-Ojeda O, Olszewska M, Qu J, Wasielewska T, He Q, Fink M, Shinglot H, Youssif M, Satter M, Wang Y, Hosey J & Quintanilla H et al (2014) Efficacy and toxicity management of 19-28z CAR T cell therapy in B cell acute lymphoblastic leukemia. *Science translational medicine* **6:** 224ra25

Fitzgerald JC, Weiss SL, Maude SL, Barrett DM, Lacey SF, Melenhorst JJ, Shaw P, Berg RA, June CH, Porter DL, Frey NV, Grupp SA & Teachey DT (2016) Cytokine Release Syndrome After Chimeric Antigen Receptor T Cell Therapy for Acute Lymphoblastic Leukemia. *Critical care medicine*

Fraietta JA, Beckwith KA, Patel PR, Ruella M, Zheng Z, Barrett DM, Lacey SF, Melenhorst JJ, McGettigan SE, Cook DR, Zhang C, Xu J, Do P, Hulitt J, Kudchodkar SB, Cogdill AP, Gill S, Porter DL, Woyach JA & Long M et al (2016) Ibrutinib enhances chimeric antigen receptor T-cell engraftment and efficacy in leukemia. *Blood* **127:** 1117–1127

Garfall AL, Maus MV, Hwang W-T, Lacey SF, Mahnke YD, Melenhorst JJ, Zheng Z, Vogl DT, Cohen AD, Weiss BM, Dengel K, Kerr NDS, Bagg A, Levine BL, June CH & Stadtmauer EA (2015) Chimeric Antigen Receptor T Cells against CD19 for Multiple Myeloma. *The New England journal of medicine* **373:** 1040–1047

Grupp SA, Kalos M, Barrett D, Aplenc R, Porter DL, Rheingold SR, Teachey DT, Chew A, Hauck B, Wright JF, Milone MC, Levine BL & June CH (2013) Chimeric antigen receptor-modified T cells for acute lymphoid leukemia. *The New England journal of medicine* **368:** 1509–1518

Guo B, Chen M, Han Q, Hui F, Dai H, Zhang W, Zhang Y, Wang Y, Zhu H & Han W (2016) CD138-directed adoptive immunotherapy of chimeric antigen receptor (CAR)-modified T cells for multiple myeloma. *Journal of Cellular Immunotherapy* **2:** 28–35

Jensen MC, Popplewell L, Cooper LJ, DiGiusto D, Kalos M, Ostberg JR & Forman SJ (2010) Antitransgene rejection responses contribute to attenuated persistence of adoptively transferred CD20/CD19-specific chimeric antigen receptor redirected T cells in humans. *Biology of blood and marrow transplantation : journal of the American Society for Blood and Marrow Transplantation* **16:** 1245–1256

Kalos M, Levine BL, Porter DL, Katz S, Grupp SA, Bagg A & June CH (2011) T cells with chimeric antigen receptors have potent antitumor effects and can establish memory in patients with advanced leukemia. *Science translational medicine* **3:** 95ra73

Kebriaei P, Singh H, Huls MH, Figliola MJ, Bassett R, Olivares S, Jena B, Dawson MJ, Kumaresan PR, Su S, Maiti S, Dai J, Moriarity B, Forget M-A, Senyukov V, Orozco A, Liu T, McCarty J, Jackson RN & Moyes JS et al (2016) Phase I trials using Sleeping Beauty to generate CD19-specific CAR T cells. *The Journal of clinical investigation* **126:** 3363–3376

Kochenderfer JN, Dudley ME, Carpenter RO, Kassim SH, Rose JJ, Telford WG, Hakim FT, Halverson DC, Fowler DH, Hardy NM, Mato AR, Hickstein DD, Gea-Banacloche JC, Pavletic SZ, Sportes C, Maric I, Feldman SA, Hansen BG, Wilder JS & Blacklock-Schuver B et al (2013) Donor-derived CD19-targeted T cells cause regression of malignancy persisting after allogeneic hematopoietic stem cell transplantation. *Blood* **122:** 4129–4139

Kochenderfer JN, Dudley ME, Feldman SA, Wilson WH, Spaner DE, Maric I, Stetler-Stevenson M, Phan GQ, Hughes MS, Sherry RM, Yang JC, Kammula US, Devillier L, Carpenter R, Nathan D-AN, Morgan RA, Laurencot C & Rosenberg SA (2012) B-cell depletion and remissions of malignancy along with cytokine-associated toxicity in a clinical trial of anti-CD19 chimeric-antigen-receptor-transduced T cells. *Blood* **119:** 2709–2720

Kochenderfer JN, Dudley ME, Kassim SH, Somerville RPT, Carpenter RO, Stetler-Stevenson M, Yang JC, Phan GQ, Hughes MS, Sherry RM, Raffeld M, Feldman S, Lu L, Li YF, Ngo LT, Goy A, Feldman T, Spaner DE, Wang ML & Chen CC et al (2015) Chemotherapy-refractory diffuse large B-cell lymphoma and indolent B-cell malignancies can be effectively treated with autologous T cells expressing an anti-CD19 chimeric antigen receptor. *Journal of clinical oncology : official journal of the American Society of Clinical Oncology* **33:** 540–549

Kochenderfer JN, Wilson WH, Janik JE, Dudley ME, Stetler-Stevenson M, Feldman SA, Maric I, Raffeld M, Nathan D-AN, Lanier BJ, Morgan RA & Rosenberg SA (2010) Eradication of B-lineage cells and regression of lymphoma in a patient treated with autologous T cells genetically engineered to recognize CD19. *Blood* **116:** 4099–4102

Lee DW, Kochenderfer JN, Stetler-Stevenson M, Cui YK, Delbrook C, Feldman SA, Fry TJ, Orentas R, Sabatino M, Shah NN, Steinberg SM, Stroncek D, Tschernia N, Yuan C, Zhang H, Zhang L, Rosenberg SA, Wayne AS & Mackall CL (2015) T cells expressing CD19 chimeric antigen receptors for acute lymphoblastic leukaemia in children and young adults: A phase 1 dose-escalation trial. *The Lancet* **385:** 517–528

Maude SL, Frey N, Shaw PA, Aplenc R, Barrett DM, Bunin NJ, Chew A, Gonzalez VE, Zheng Z, Lacey SF, Mahnke YD, Melenhorst JJ, Rheingold SR, Shen A, Teachey DT, Levine BL, June CH, Porter DL & Grupp SA (2014) Chimeric antigen receptor T cells for sustained remissions in leukemia. *N. Engl. J. Med* **371:** 1507–1517

Porter DL, Hwang W-T, Frey NV, Lacey SF, Shaw PA, Loren AW, Bagg A, Marcucci KT, Shen A, Gonzalez V, Ambrose D, Grupp SA, Chew A, Zheng Z, Milone MC, Levine BL, Melenhorst JJ & June CH (2015) Chimeric antigen receptor T cells persist and induce sustained remissions in relapsed refractory chronic lymphocytic leukemia. *Science translational medicine* **7:** 303ra139

Porter DL, Levine BL, Kalos M, Bagg A & June CH (2011) Chimeric antigen receptor-modified T cells in chronic lymphoid leukemia. *The New England journal of medicine* **365:** 725–733

Ramos CA, Savoldo B, Torrano V, Ballard B, Zhang H, Dakhova O, Liu E, Carrum G, Kamble RT, Gee AP, Mei Z, Wu M-F, Liu H, Grilley B, Rooney CM, Brenner MK, Heslop HE & Dotti G (2016) Clinical responses with T lymphocytes targeting malignancy-associated kappa light chains. *The Journal of clinical investigation*

Ritchie DS, Neeson PJ, Khot A, Peinert S, Tai T, Tainton K, Chen K, Shin M, Wall DM, Honemann D, Gambell P, Westerman DA, Haurat J, Westwood JA, Scott AM, Kravets L, Dickinson M, Trapani JA, Smyth MJ & Darcy PK et al (2013) Persistence and efficacy of second generation CAR T cell against the LeY antigen in acute myeloid leukemia. *Molecular therapy : the journal of the American Society of Gene Therapy* **21:** 2122–2129

Savoldo B, Ramos CA, Liu E, Mims MP, Keating MJ, Carrum G, Kamble RT, Bollard CM, Gee AP, Mei Z, Liu H, Grilley B, Rooney CM, Heslop HE, Brenner MK & Dotti G (2011) CD28 costimulation improves expansion and persistence of chimeric antigen receptor-modified T cells in lymphoma patients. *The Journal of clinical investigation* **121:** 1822–1826

Till BG, Jensen MC, Wang J, Chen EY, Wood BL, Greisman HA, Qian X, James SE, Raubitschek A, Forman SJ, Gopal AK, Pagel JM, Lindgren CG, Greenberg PD, Riddell SR & Press OW (2008) Adoptive immunotherapy for indolent non-Hodgkin lymphoma and mantle cell lymphoma using genetically modified autologous CD20-specific T cells. *Blood* **112:** 2261–2271

Till BG, Jensen MC, Wang J, Qian X, Gopal AK, Maloney DG, Lindgren CG, Lin Y, Pagel JM, Budde LE, Raubitschek A, Forman SJ, Greenberg PD, Riddell SR & Press OW (2012) CD20-specific adoptive immunotherapy for lymphoma using a chimeric antigen receptor with both CD28 and 4-1BB domains: pilot clinical trial results. *Blood* **119:** 3940–3950

Turtle CJ, Hanafi L-A, Berger C, Gooley TA, Cherian S, Hudecek M, Sommermeyer D, Melville K, Pender B, Budiarto TM, Robinson E, Steevens NN, Chaney C, Soma L, Chen X, Yeung C, Wood B, Li D, Cao J & Heimfeld S et al (2016) CD19 CAR-T cells of defined CD4+:CD8+ composition in adult B cell ALL patients. *The Journal of clinical investigation* **126:** 2123–2138

Wang C, Wu Z, Wang Y, Guo Y, Dai H, Wang X-H, Li X, Zhang Y-j, Zhang W-y, Chen M-x, Zhang Y, Feng K-c, Liu Y, Li S-X, Yang Q-M & Han W (2016a) Autologous T cells expressing CD30 chimeric antigen receptors for relapsed or refractory Hodgkin’s lymphoma: an open-label phase I trial. *Clinical cancer research : an official journal of the American Association for Cancer Research*

Wang Q-s, Wang Y, Lv H-y, Han Q-w, Fan H, Guo B, Wang L-l & Han W-d (2015) Treatment of CD33-directed chimeric antigen receptor-modified T cells in one patient with relapsed and refractory acute myeloid leukemia. *Molecular therapy : the journal of the American Society of Gene Therapy* **23:** 184–191

Wang X, Popplewell LL, Wagner JR, Naranjo A, Blanchard MS, Mott MR, Norris AP, Wong CW, Urak RZ, Chang W-C, Khaled SK, Siddiqi T, Budde LE, Xu J, Chang B, Gidwaney N, Thomas SH, Cooper LJN, Riddell SR & Brown CE et al (2016b) Phase 1 studies of central memory-derived CD19 CAR T-cell therapy following autologous HSCT in patients with B-cell NHL. *Blood* **127:** 2980–2990

Wang Y, Zhang W-y, Han Q-w, Liu Y, Dai H-r, Guo Y-l, Bo J, Fan H, Zhang Y, Zhang Y-j, Chen M-x, Feng K-c, Wang Q-s, Fu X-b & Han W-d (2014) Effective response and delayed toxicities of refractory advanced diffuse large B-cell lymphoma treated by CD20-directed chimeric antigen receptor-modified T cells. *Clinical immunology (Orlando, Fla.)* **155:** 160–175

1. [?] Data from 29 CAR T cell clinical trials for haematological malignancies published by the end of 2016 were collected. In the first column, the targeted antigen, the antibody clone of the scFv and the treated indication are listed. The second column provides information about the T cell origin and whether stem cell transplantation, a pre-treatment like lymphodepletion, or chemotherapy, or systemic IL-2 administration were applied. In the third column the domain organisation of the CAR construct, the used vector to generate CAR T cells as well as the percentage of generated CAR T cells are mentioned. In the next column information about the applied T cell dose and number of infusions is provided as well as information on of CAR T cells persistence (longest observed time frame in single patients) and the applied detection method. In addition, information on the number, age and clinical outcome of treated patient as well as the phase of the trial and observed toxicities are provided. In the last column, the trial identifier, publication reference and the status of the trial (ongoing, completed; suspended; unknown) are indicated. Information about disease status is provided in further footnotes. [↑](#footnote-ref-2)
2. [?] Targeting of MRD after hematopoietic stem cell transplantation in patients with multiply relapsed B cell NHL. [↑](#footnote-ref-3)
3. [?] Targeting of MRD after hematopoietic stem cell transplantation in patients with advanced-stage disease (multiply relapsed B-lineage ALL or B cell NHL). [↑](#footnote-ref-4)
4. [?] Six patients had detectable leukemic cells (n=4) or MRD (n=2) at the time of the CAR T cell infusion. Two patients were in remission at the time of CAR T cell infusion but at a high risk for relapse. Both patients in remission achieved a continuing complete response. The two patients with MRD had a complete response and stable disease as best clinical outcome, respectively. [↑](#footnote-ref-5)
5. [?] Two patients were in partial remission after most recent standard donor lymphocyte infusion before CAR T cell infusion and reached stable disease (SD) as best clinical outcome. [↑](#footnote-ref-6)
6. [?] The clinical trial included patients with absent minimal residual disease (n=5), detectable disease (n=24) and morphologic remission (n=1). [↑](#footnote-ref-7)
7. [?] At baseline disease assessment, 3 (38%) participants had a complete response (CR), 2 (25%) had a partial response (PR), 2 (25%) had stable disease (SD), and 1 (13%) had failed primary induction with stable disease after salvage therapy at transplant. [↑](#footnote-ref-8)
8. [?] The median leukemia burden was 26% marrow blast of mononuclear cells (0.03-96%) before CAR T cell infusion. Six patients had never attained an MRD-negative remission and two patients had measurable CNS leukemia. No difference in the best clinical outcome dependent on the baseline disease assessment could be observed. [↑](#footnote-ref-9)
9. [?]The clinical trial included patients with minimal residual disease (<5% blasts; n=10) and patients with morphologic disease (>5% blasts; n=20). [↑](#footnote-ref-10)
10. [?] At baseline disease assessment, 6 (75%) participants had a complete response (CR) and 2 (25%) had a partial response (PR). [↑](#footnote-ref-11)
11. [?] The clinical trial included patients with minimal residual disease (n=7) and morphologic residual disease (n=9). No differences in the clinical outcomes of patients with MRD versus those patients with overt morphologic residual leukemia were observed. [↑](#footnote-ref-12)
12. [?] One patient had partial remission at baseline disease assessment and achieved a complete response as best clinical outcome. [↑](#footnote-ref-13)
13. [?] The treated patients had stage II to IV relapsed or refractory indolent B-cell NHL or MCL. After cytoreductive chemotherapy two patients achieved already complete response and ended up with no evidence of disease after CAR T cell infusion. [↑](#footnote-ref-14)
