# Supplementary material for: Clinical development of CAR T cells—challenges and opportunities in translating innovative treatment concepts
Source: EMBO Mol Med. 2017 Aug 1;9(9):1183–97. doi: 10.15252/emmm.201607485 (PMC5582407; doi:10.15252/emmm.201607485)
Supplement: Supplementary file 6 — Dataset EV5 [file EMMM-9-1183-s006.doc]

## Dataset EV5. Active and non-active CAR gene therapy clinical trials in Europe (19 total)

| **Status** | **Phase** | **Identifier/Ref.** | **Antigen** | **Titel** | **Sponsor** | **Country** |
| --- | --- | --- | --- | --- | --- | --- |
| T | 1/2 | DDHK97-29/P00.0040C[[[1]](#footnote-2)] | CAIX | *NA* | EUMC | EU (NL) |
| T | 1 | NCT01212887 | CEA | Treated Blood Cells, Cyclophosphamide, Fludarabine Phosphate, and Aldesleukin in Treating Patients With Cancer | CRUK | EU (UK) |
| T | 1/2 | NCT01195480 | CD19 | CD19-CAR Immunotherapy for Childhood Acute Lymphoblastic Leukemia (ALL) | UCL | EU (UK, DE) |
| O | 1 | NCT02431988 | CD19 | Evaluation of CAR19 T-cells as an Optimal Bridge to Allogeneic Transplantation | UCL | EU (UK) |
| O | 1 | NCT02443831 | CD19 | CARPALL: Immunotherapy With CD19 CAR T-cells for CD19+ Haematological Malignancies | UCL | EU (UK) |
| O | 1/2 | NCT02132624 | CD19 | CD19-targeting 3rd Generation CAR T Cells for Refractory B Cell Malignancy - a Phase I/IIa Trial. | UU | EU (SE) |
| O | 1 | NCT01493453 | CD19 | A Phase I Study of CD19 Specific T Cells in CD19 Positive Malignancy | CHNHS | EU (UK) |
| O | 2 | NCT02445248 | CD19 | Study of Efficacy and Safety of CTL019 in Adult DLBCL Patients | Novartis | USA ,CA, AU, JP, EU (DE, AT, FR, IT, NL, NO) |
| O | 1 | NCT02746952 | CD19 | Dose Escalation Study to Evaluate the Safety, Tolerability and Biological Activity of a Single Dose of UCART19 in Patients With Relapsed / Refractory B-cell ALL and CLL | KCH | EU (UK) |
| O | 1 | NCT02808442 | CD19 | Study of UCART19 in Pediatric Patients With Relapsed/Refractory B Acute Lymphoblastic Leukemia | IRIS | EU (UK) |
| O | 2 | NCT02435849 | CD19 | Determine Efficacy and Safety of CTL019 in Pediatric Patients With Relapsed and Refractory B-cell ALL | Novartis | USA, CA, AU, JP, EU (AT, BE, FR, DE, IT, NO, ES) |
| O | 1 | NCT02935257 | CD19 | Immunotherapy for High Risk/Relapsed CD19+ Acute Lymphoblastic Leukaemia Using CAR T-cells to Target CD19 | UCL | EU (UK) |
| O | 1 | NCT01645293 | CD30 | Multicenter Phase I Trial of Engineered T Cells for Patients With Relapsed or Refractory Primary Cutaneous CD30+ Large T Cell Lymphoma or Transformed CD30+ Mycosis Fungoides | UC | EU (DE) |
| O | 1 | NCT01818323 | ErbB2/  Her2 | Phase I Trial: T4 Immunotherapy of Head and Neck Cancer | KCL | EU (UK) |
| O | 1 | NCT01722149 | FAP | Re-directed T Cells for the Treatment (FAP)-Positive Malignant Pleural Mesothelioma | ZU | EU (CH) |
| O | 1 | NCT02761915 | GD2 | A Cancer Research UK Trial of Anti-GD2 T-cells (1RG-CART) | CRUK | EU (UK) |
| O | *NA* | NCT02842320 | IL-1RAP | Targeting Leukemic Stem Cell Expressing the IL-1RAP Protein in Chronic Myelogenous Leukemia (CML) | CHUB | EU (FR) |
| O | LFU | NCT02445222 | CD19 | CD19 CART Long Term Follow-up (LTFU) Study | Novartis | USA, CA, AU, EU (ES, AT) |
| O | LFU | NCT02735083 | CD19 | A Study to Evaluate the Long-term Safety of Patients With Advanced Lymphoid Malignancies Who Have Been Previously Administered With UCART19 | IRIS | EU (UK) |

***NA***, not available, **O**, ongoing; **T**, terminated; **LFU**, long-term follow-up; **CAIX**, carbonic anhydrase IX; **CEA**, carcinoembryonic antigen; **ErbB2/Her2**, human epidermal growth factor receptor 2; **FAP**, Fibroblast activation protein; **IL-1RAP**, Interleukin-1 receptor accessory protein; **CHNHS**, Christie Hospital NHS Foundation Trust; **CHUB**, Centre Hospitalier Universitaire de Besancon; **CRUK**, Cancer Research UK; **EUMC**, Erasmus University Medical Center; **IRIS**, Institut de Recherches Internationales Servier; **KCH**, King's College Hospital NHS Trust; **KCL**, King's College London; **Novartis**, Novartis Pharmaceuticals; **UC**, University of Cologne; **UCL**, University College, London; **UU**, Uppsala University; **ZU**, University of Zurich; **AT**, Austria; **AU**, Australia; **BE**, Belgium; **CA**, Canada; **CH**, Switzerland; **DE**, Germany; **ES**, Spain; **EU**, Europe; **FR**, France; **IT**, Italy; **JP**, Japan; **NL**, Netherlands; **NO**, Norway; **SE**, Sweden; **UK**, United Kingdom; **USA**, United States of America.

1. [?] Lamers CH, Sleijfer S, van Steenbergen S, van Elzakker P, van Krimpen B, Groot C, Vulto A, den Bakker M, Oosterwijk E, Debets R & Gratama JW (2013) Treatment of metastatic renal cell carcinoma with CAIX CAR-engineered T cells: clinical evaluation and management of on-target toxicity. *Molecular therapy : the journal of the American Society of Gene Therapy* **21:** 904–912 [↑](#footnote-ref-2)
